# Unconventional genetic code systems in archaea

**DOI:** 10.3389/fmicb.2022.1007832

**Published:** 2022-09-08

**Authors:** Kexin Meng, Christina Z. Chung, Dieter Söll, Natalie Krahn

**Affiliations:** ^1^Department of Molecular Biophysics & Biochemistry, Yale University, New Haven, CT, United States; ^2^Department of Chemistry, Yale University, New Haven, CT, United States

**Keywords:** archaea, selenocysteine, pyrrolysine, phosphoserine, genetic code expansion

## Abstract

Archaea constitute the third domain of life, distinct from bacteria and eukaryotes given their ability to tolerate extreme environments. To survive these harsh conditions, certain archaeal lineages possess unique genetic code systems to encode either selenocysteine or pyrrolysine, rare amino acids not found in all organisms. Furthermore, archaea utilize alternate tRNA-dependent pathways to biosynthesize and incorporate members of the 20 canonical amino acids. Recent discoveries of new archaeal species have revealed the co-occurrence of these genetic code systems within a single lineage. This review discusses the diverse genetic code systems of archaea, while detailing the associated biochemical elements and molecular mechanisms.

## Introduction

In early taxonomic classifications, archaea and bacteria were grouped together given their shared prokaryotic nature under the taxon ‘Monera’ (Whittaker, 1969). However, sequence and phylogenetic analyses of the universal small ribosomal RNA (rRNA) led to the recognition of archaea as an independent third domain of life, alongside eukaryotes and bacteria (Woese et al., 1975). Further study of archaea revealed many similarities in their molecular mechanisms compared to eukaryotes, now suggesting a closer evolutionary history between these two domains of life.

Like eukaryotes and bacteria, archaea inhabit a wide range of environments, from soil to the human gut (Baker et al., 2020). However, the archaeal domain is distinct for including many methanogens and extremophiles that can survive in environments with pH, temperature, salt concentration, and pressure outside standard conditions (Rothschild and Mancinelli, 2001). Given the extreme environments archaea inhabit, certain archaeal lineages also possess unique genetic code systems that are not as prominent in organisms of other domains. This is necessary for the formation of enzymes that function in niche pathways, such as methanogenesis, or are more suited for survival in harsh conditions. The diverse genetic code systems seen in archaea include the natural recoding of stop codons for insertion of the 21st and 22nd genetically encoded amino acids (selenocysteine (Sec) and pyrrolysine (Pyl), respectively), and tRNA-dependent pathways for canonical amino acid biosynthesis. Recent studies have uncovered archaeal lineages, recovered from geothermal springs and deep-sea sediments, which possess more than one of these diverse genetic code systems (Mukai et al., 2017; Sun et al., 2021). Here, we discuss the structural elements, regulatory mechanisms, and biosynthetic strategies associated with these systems.

## Selenocysteine insertion

Selenocysteine, the 21st genetically encoded amino acid, was discovered in 1976 (Cone et al., 1976) and later shown to be encoded by an opal (UGA) stop codon (Zinoni et al., 1986; Garcia and Stadtman, 1992). Sec is chemically similar to cysteine (Cys), except with a selenol group in place of the thiol group, resulting in the high nucleophilicity of Sec (Stock and Rother, 2009). The selenol group is also deprotonated at lower pH values in comparison to the thiol group, leading Sec to be more reactive than Cys and commonly present in the catalytic site of redox enzymes. Sec is found in all three domains of life with the majority of Sec-encoding archaea being methanogens. Methanogens are highly reliant on selenium for growth as the majority of archaeal selenoproteins are involved in methanogenesis (Rother et al., 2001). These selenoproteins include heterodisulfide reductases, dehydrogenases, and hydrogenases (Rother and Quitzke, 2018). The Sec residue in heterodisulfide reductases ligates to iron–sulfur clusters and is thought to guide conformational changes. In dehydrogenases, Sec functions in the catalytic center to coordinate cofactors. Sec is also found in Ni-containing hydrogenases, coordinating the Ni with three additional Cys residues. In these [NiFeSe]-hydrogenases, Sec is responsible for incorporating Ni into the active site and increasing tolerance to oxidative stress. In comparison to [NiFe]-hydrogenases with four active-site Cys residues, [NiFeSe]-hydrogenases have higher enzymatic activity (Evans et al., 2021). However, when active-site Cys residues are substituted with Sec, oxidative stress tolerance is significantly increased, but enzymatic activity is reduced, as selenium disrupts the proton transfer pathway (Evans et al., 2021). These findings suggest Sec is incorporated in a specific functional context and is not necessarily catalytically superior to Cys in all circumstances. In addition to methanogenesis, Sec is also found in enzymes of the selenoprotein biosynthesis pathway, as well as in HesB-like and peroxiredoxin-like proteins of unknown functions (Rother and Quitzke, 2018). Thus, further exploration is needed to elucidate the different functions of archaeal selenoproteins.

Recently, genomic analysis revealed the complete set of known archaeal Sec-encoding genes in the newly discovered archaeal phyla *Lokiarchaeota* and *Candidatus* Sifarchaeia. Both *Lokiarchaeota* and Sifarchaeia belong to the non-methanogenic Asgardarchaeota superphylum (Sun et al., 2021). Phylogenetic reconstructions posit *Lokiarchaeota* to be a sister group of eukaryotes. Based on the unique characteristics in parts of the Sec biosynthesis and insertion pathways, it is suggested that this phylum is a potential evolutionary connection between archaea and eukaryotes (Mariotti et al., 2016).

**Figure 1:**
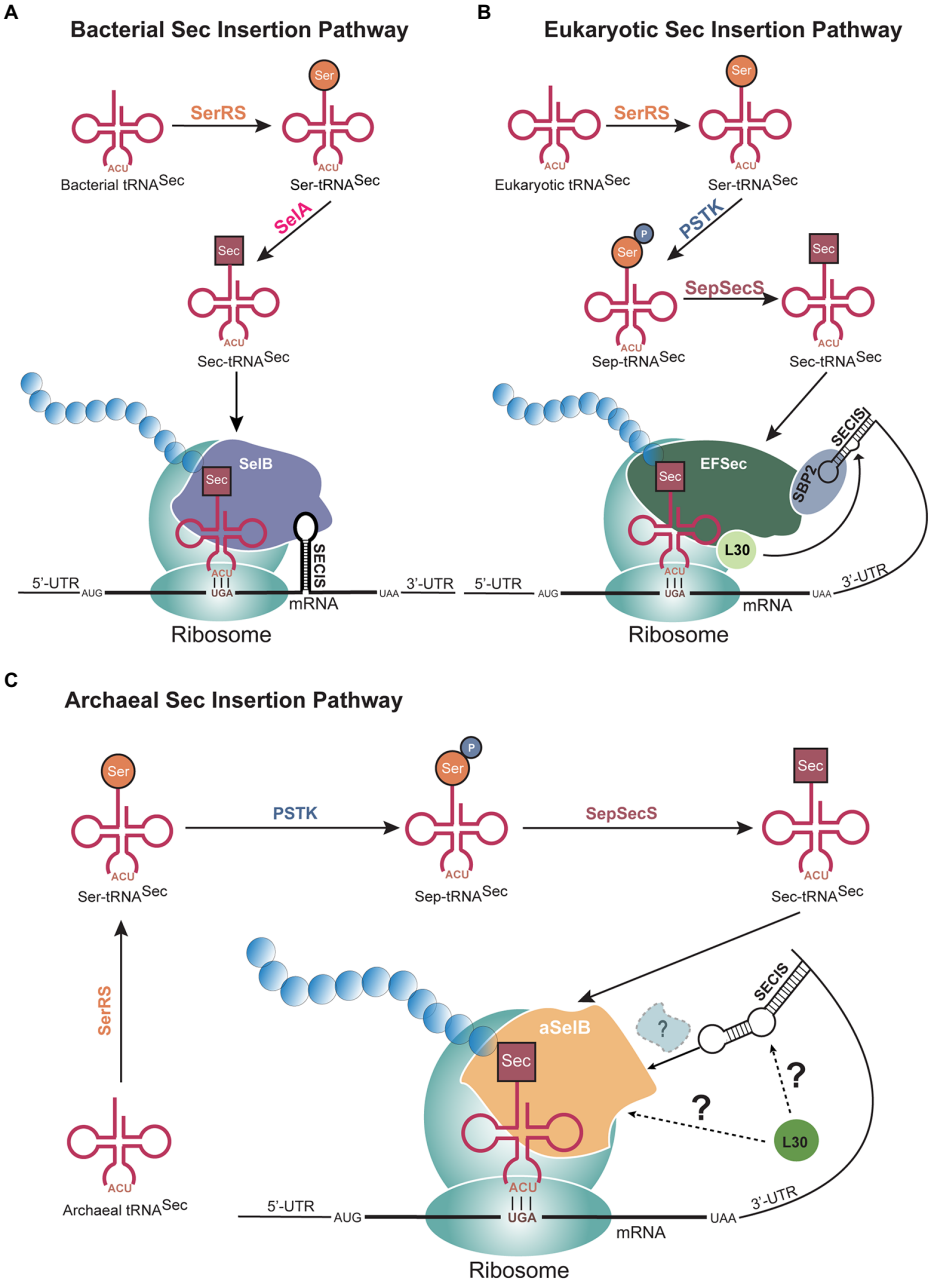
Selenocysteine (Sec) biosynthesis pathways in all domains of life. **(A)** In bacteria, the conversion of serine (Ser) to Sec occurs through a single step catalyzed by selenocysteine synthase (SelA), after tRNA^Sec^ is aminoacylated by seryl-tRNA synthetase (SerRS). Sec is then incorporated at UGA codons with an immediate downstream selenocysteine insertion sequence (SECIS) element. In **(B)** eukaryotes and **(C)** archaea, two steps are needed to synthesize Sec from Ser, through the activity of phosphoseryl-tRNA^Sec^ kinase (PSTK) and *O*-phosphoseryl-tRNA^Sec^:Sec synthase (SepSecS). The 3′-untranslated region (UTR), which contains the SECIS element, bends to position the mRNA element closer to the UGA codon. The factors mediating this recruitment are not fully known in archaea and eukaryotes. In all three domains, a Sec-specific elongation factor (SelB in bacteria, aSelB in archaea, and EFSec in eukaryotes) functions to bring Sec-tRNA^Sec^ to the ribosome.

Although Sec is found in all domains of life, there are some differences in the mechanism of Sec biosynthesis and incorporation (Figure 1). The initial step is conserved across all domains: aminoacylation of a Sec-specific tRNA isoacceptor (tRNA^Sec^) with serine (Ser) by seryl-tRNA synthetase (SerRS; Rother and Quitzke, 2018). It is at the Ser to Sec conversion where the bacterial mechanism splits from archaea and eukaryotes. In bacteria, this conversion occurs in a single step, after which the bacterial Sec-specific elongation factor SelB transports selenocysteinyl-tRNA^Sec^ (Sec-tRNA^Sec^) to the ribosome (Figure 1A). In archaea and eukaryotes, two separate enzymes are needed to convert Ser to Sec (Figures 1B,C). The Ser is first phosphorylated to phosphoserine (Sep) by phosphoseryl-tRNA^Sec^ kinase (PSTK) in an ATP-dependent reaction, converting Ser-tRNA^Sec^ to Sep-tRNA^Sec^ (Carlson et al., 2004; Sherrer et al., 2008b). Sep is subsequently converted to Sec by *O*-phosphoseryl-tRNA^Sec^:Sec synthase (SepSecS) in a pyridoxal phosphate-dependent manner. The resulting Sec-tRNA^Sec^ is then brought to the ribosome to decode a UGA codon. At this point, the detailed elongation mechanism is not fully understood in either eukaryotes or archaea. Current information indicates that a specialized elongation factor (EFSec in eukaryotes and aSelB, also known as aEFSec, in archaea), recognizes a Sec insertion sequence (SECIS) element in the 3′-untranslated region (UTR) of the mRNA in addition to the Sec-tRNA^Sec^, to recode UGA. In eukaryotes, it is believed the 3′-UTR, containing the SECIS element, wraps around to position the SECIS element more closely to the UGA codon (Figure 1B). This process does not occur in bacteria as the bacterial SECIS is located in the translated region, immediately downstream of the UGA codon (Kinzy et al., 2005; Figure 1A). It is hypothesized that Sec insertion in archaea follows a similar process to that in eukaryotes since the SECIS element is situated in the same region (Figure 1C).

In addition to the proteins directly involved in the Sec biosynthesis pathway, the archaeal genome also encodes a selenium-binding protein (SeBP; Self et al., 2004; Patteson et al., 2005). The tetrameric SeBP binds one molecule of reduced selenium. Though the exact role of SeBP has yet to be elucidated, these proteins are hypothesized to transport cytosolic selenium to archaeal selenophosphate synthetase, a homolog of the bacterial *selD* gene (Self et al., 2004). Selenophosphate synthetase then converts reduced selenium to selenomonophosphate, the selenium donor for conversion of Sep to Sec. The complex Sec biosynthesis and translation pathways are facilitated by the distinct features of tRNA^Sec^ structure and the presence of a SECIS element.

### tRNA^Sec^ structure

**Figure 2:**
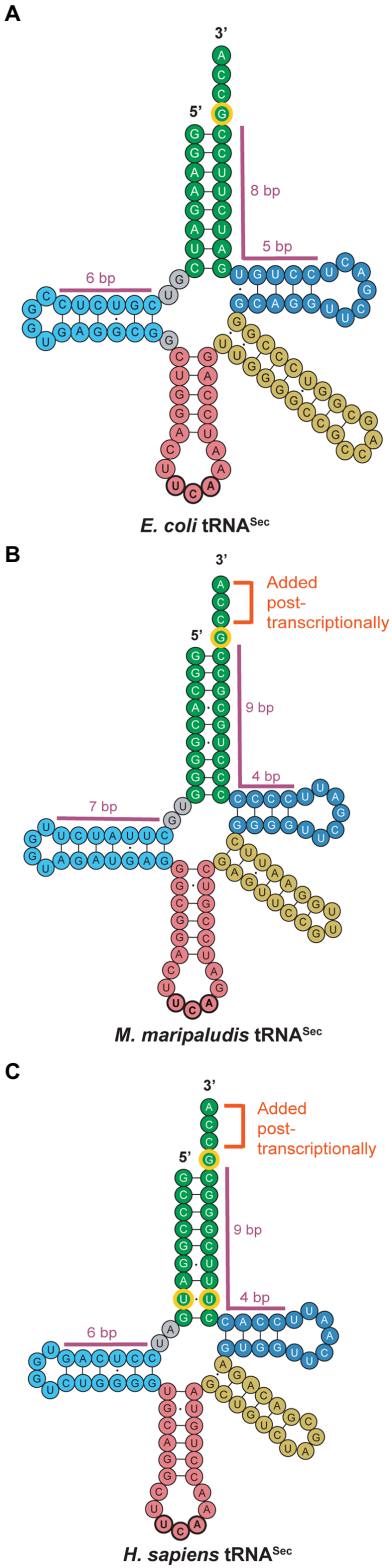
tRNA^Sec^ secondary structures across all domains of life. Small but conserved differences are observed between **(A)** bacterial, **(B)** archaeal, and **(C)** eukaryotic tRNA^Sec^. While canonical archaeal and eukaryotic tRNA^Sec^ share the post-transcriptionally added CCA tail and the 9/4 configuration of the acceptor stem (green) and T-arm (dark blue), archaeal tRNA^Sec^ possesses a longer D-arm (light blue) than both bacterial and eukaryotic tRNA^Sec^. The tRNA^Sec^ for all three domains share the G73 discriminator base as an identity element (outlined in yellow), but eukaryotic tRNA^Sec^ also has a conserved U6:U67 base pair. Note that exceptions to the canonical archaeal tRNA^Sec^ shown in this figure have been reported.

The structure of tRNA^Sec^ is the key mediator for individual reactions of Sec biosynthesis and insertion into a polypeptide. In contrast to the structure of canonical tRNAs with a 12 bp acceptor domain (acceptor stem and T-stem combined), tRNA^Sec^ has a 13 bp acceptor domain (Sturchler et al., 1993; Figure 2). This major distinguishing feature of tRNA^Sec^, prevents recognition by the general elongation factor (EF-Tu or EF-1α) and enables elongation by SelB or EFSec. In archaea, this 13 bp acceptor domain is found in a 9/4 configuration, with 9 bp in the acceptor stem and 4 bp in the T-stem (Figure 2B). This same configuration occurs in eukaryotes (Figure 2C), whereas in bacteria, tRNA^Sec^ adopts an 8/5 configuration (Schön et al., 1989; Itoh et al., 2009; Figure 2A). The 13 bp acceptor domain also serves as a recognition element for PSTK and SepSecS in archaea and eukaryotes and is needed for recognition by SelA in bacteria, for conversion of Ser to Sec.

Another unique feature of tRNA^Sec^ is its long D-stem and small D-loop (Figure 2). In archaea, the D-arm has a 7 bp stem with a 4 base loop (Figure 2B), differing from eukaryotic and bacterial tRNA^Sec^ which have a 6 bp stem and 4 base loop (Figures 2A,C). Currently, the only known exceptions to this 7 bp D-stem configuration in archaea are in the *Methanopyrus kandleri* species and Sifarchaeia phylum which encode tRNA^Sec^ with a 6 bp D-stem (Sherrer et al., 2010). Moreover, while the D-stem is important for recognition by SelA and PSTK in bacteria and eukaryotes, respectively, binding of the D-arm with the C-terminal domain (CTD) of PSTK in archaea only plays a minor role in recognition. Instead, the major contributor to the interaction between tRNA^Sec^ and PSTK in archaea is the binding of the 13 bp acceptor domain to the N-terminal domain (NTD) of PSTK (Sherrer et al., 2008a). The G73 discriminator base is conserved in all tRNA^Sec^ for recognition by SerRS and interacts with residues of PSTK to promote phosphorylation of Ser-tRNA^Sec^. Furthermore, the CCA tail of tRNA^Sec^ is not encoded in the archaeal genome, as commonly observed in bacteria, but is rather added post-transcriptionally by a CCA-adding enzyme, similar to eukaryotes (Santesmasses et al., 2017).

Though the structure of tRNA^Sec^ is highly conserved in most archaea, there are certain organisms with unique features. Notably, the *Lokiarchaeota* lineage contains the only archaeal case of a tRNA^Sec^ gene with an intron (Santesmasses et al., 2017). The intron in *Lokiarchaeota* tRNA^Sec^ is 31 nucleotides in length and located in the T-arm, an intron position previously observed in canonical archaeal tRNAs (Mariotti et al., 2016). The eukaryote species, *Daphnia pulex*, is the only other occurrence annotated thus far of an intron encoded within both of its tRNA^Sec^ genes (Santesmasses et al., 2017). The tRNA^Sec^ of *Candidatus* Bathyarchaeota also has a unique feature that is not characteristic of archaea. Its tRNA^Sec^ has a U6:U67 mismatch (Mukai et al., 2021) which is a conserved feature of eukaryotic tRNA^Sec^ (Figure 2C). These shared features between eukaryotic tRNA^Sec^ with *Lokiarchaeota* and Bathyarchaeota suggest evolution of the eukaryotic Sec pathway from them.

### Regulation of Sec insertion

Organisms have evolved a highly sophisticated method to differentiate between UGA stop codons and UGA codons signaling Sec insertion. The main distinguishing factor is the presence of an mRNA hairpin known as the SECIS element, which is bound by SelB in bacteria and is believed to form a complex with additional proteins in archaea and eukaryotes (Leibundgut et al., 2005). Bacterial SelB binds the SECIS element through a C-terminal extension (domain IV, 24 kDa), not found in EF-Tu (Selmer and Su, 2002). In eukaryotes, domain IV of EFSec is significantly shorter than its bacterial homolog, and it follows that EFSec does not bind the SECIS element directly (Zavacki et al., 2003). Instead, EFSec interacts with SECIS-binding protein 2 (SBP2) to facilitate recognition of the SECIS element. In comparison to EFSec, aSelB possesses an even shorter domain IV (8 kDa) and also fails to bind the SECIS element (Rother et al., 2001; Leibundgut et al., 2005). SelB, aSelB, and EFSec demonstrate extensive homology with the general elongation factors, containing four of the five guanosine triphosphate-binding domains found in EF-Tu/EF-1α (Fagegaltier et al., 2001). When compared to their corresponding general elongation factors, SelB, aSelB, and EFSec also contain four deletions of variable sizes in the common domain, with a fifth deletion in the aSelB of *Methanococcus jannaschii*. However, there is little sequence similarity between the CTD of SelB, aSelB, and EFSec, except for a single block of significant sequence conservation in domain IV of aSelB and EFSec (Fagegaltier et al., 2000). These similarities between the sequences and the properties of aSelB and EFSec further support the hypothesis that the archaeal Sec insertion mechanism resembles that of eukaryotes, suggesting the direct recognition between bacterial SelB and the SECIS element evolved into a mediated interaction in archaea and eukaryotes (Fagegaltier et al., 2001).

The SECIS element in archaea resides in the 3′-UTR of the mRNA (Wilting et al., 1997). Archaeal selenoprotein genes encode either one or two Sec residues, though only a single SECIS element has been found in each gene (Rother et al., 2000). This suggests one SECIS element functions to insert Sec at two different UGA codons within the same gene. The observed distance between the SECIS element and the Sec codons for which it regulates ranges between 70 and 1,500 nucleotides (Rother and Quitzke, 2018). One exception is the SECIS element for the *fdhA* gene in *M. jannaschii*. In this case, the SECIS element is in the 5′-UTR, 450 nucleotides upstream of its cognate Sec codon (Wilting et al., 1997; Rother and Quitzke, 2018). It is hypothesized that this is due to a distance constraint with the 3′-UTR being over 1,600 nucleotides away from the Sec codon. Therefore, with the SECIS element located in the 5′-UTR, closer to the Sec codon, it can bend in similar manner as what is suggested for the 3′-UTR to regulate Sec insertion.

Though the SECIS structure is highly conserved, the sequence is quite variable among selenoproteins and organisms, suggesting no distinct evolutionary origin. It should be noted that most archaeal SECIS elements have two apical loops, which is distinct from the singular apical loop in eukaryotic and bacterial SECIS elements (Figures 1B,C). However, recent work investigating these structures revealed that the SECIS element in *Lokiarchaeota* has only one apical loop (Mariotti et al., 2016) and shares similar sequence elements to those in eukaryotes (Latrèche et al., 2009; Mariotti et al., 2016). Since *Lokiarchaeota* express similar selenoproteins to those in other archaea, the differing features of the SECIS element further enhance the theory that *Lokiarchaeota* may be the connection between archaea and eukaryotes. Moreover, the discovery of *Lokiarchaeota* supports the idea that the ability to encode Sec was introduced into eukaryotes from archaea through vertical gene transfer (Rother and Quitzke, 2018).

While SBP2 facilitates eukaryotic Sec insertion by recruiting the SECIS element to the UGA codon, no protein in archaea has yet been identified to be involved in binding to the SECIS element (Rother and Quitzke, 2018). However, homologs to the ribosomal protein L30 are encoded in *M. jannaschii* and *Methanococcus maripaludis* (Bult et al., 1996; Poehlein et al., 2018). L30 in eukaryotes binds the kink-turn structure of the eukaryal SECIS element and is thought to trigger insertion of Sec-tRNA^Sec^ into the ribosomal A site (Rother and Quitzke, 2018). Ascertainment of the function of archaeal homologs of L30 in selenoprotein production has yet to be conducted as the closest homolog of L30 in *M. maripaludis* was shown to be essential, thus preventing the study of its function through using mutational analysis. Additionally, *in vitro* assays showed that purified L30 from *M. maripaludis* does not bind the archaeal SECIS element. Thus, the mechanism by which SelB, the ribosome, and the SECIS element communicate in archaea to insert Sec for recoding of the UGA codon remains unknown. Current research still needs to address the question of whether there are other potential binding proteins encoded in the archaeal genome that have yet to be discovered.

## Pyrrolysine insertion

Genetic encoding of pyrrolysine was first discovered in the methylamine methyltransferase of *Methanosarcina barkeri* in 2002 (Hao et al., 2002; Srinivasan et al., 2002). Since then, most archaea currently known to encode Pyl fall into two methanogen families: *Methanosarcinaceae* and *Methanomassiliicoccus* (Gaston et al., 2011a; Borrel et al., 2014). However, recent discovery of the non-methanogenic lineage, *Candidatus* Sifarchaeia, indicated that Pyl is not exclusive to methanogens (Sun et al., 2021). The amino acid Pyl is biosynthesized by enzymes encoded through the *pylB*, *pylC*, and *pylD* genes (Gaston et al., 2011b). In contrast to the Sec biosynthesis and insertion pathway, Pyl is not synthesized in a tRNA-dependent manner. Instead, like canonical amino acids, there is a dedicated tRNA isoacceptor (tRNA^Pyl^) and aminoacyl-tRNA synthetase (pyrrolysyl-tRNA synthetase, PylRS) for insertion of Pyl at specific UAG (amber) codons (Blight et al., 2004).

In the genome of the *Methanosarcinaceae*, the *pyl* genes are present in an uninterrupted cluster as *pylTSBCD*, with *pylT* and *pylS* encoding tRNA^Pyl^ and PylRS, respectively (Borrel et al., 2014). *Methanohalobium evestigatum* is an exception, containing two separate genomic clusters, *pylTS* and *pylBCD* (Gaston et al., 2011a). In the *Methanomassiliicoccus* family, the *pyl* genes are organized in a manner distinct from that of *Methanosarcinaceae* and from each other. In *Candidatus* Methanomethylophilus alvus Mx1201, the *pyl* genes are encoded as *pylTSCD*, with *pylB* separated from the cluster (Borrel et al., 2012). *Methanomassiliicoccus luminyensis* possesses two copies of the *pylTSBCD* cluster with an additional third isolated copy of *pylT* (Borrel et al., 2014). The difference between the Pyl systems of *Methanosarcinaceae* and *Methanomassiliicoccus* is further reflected in the structures of their PylRS enzymes, discussed below.

### PylRS

**Figure 3:**
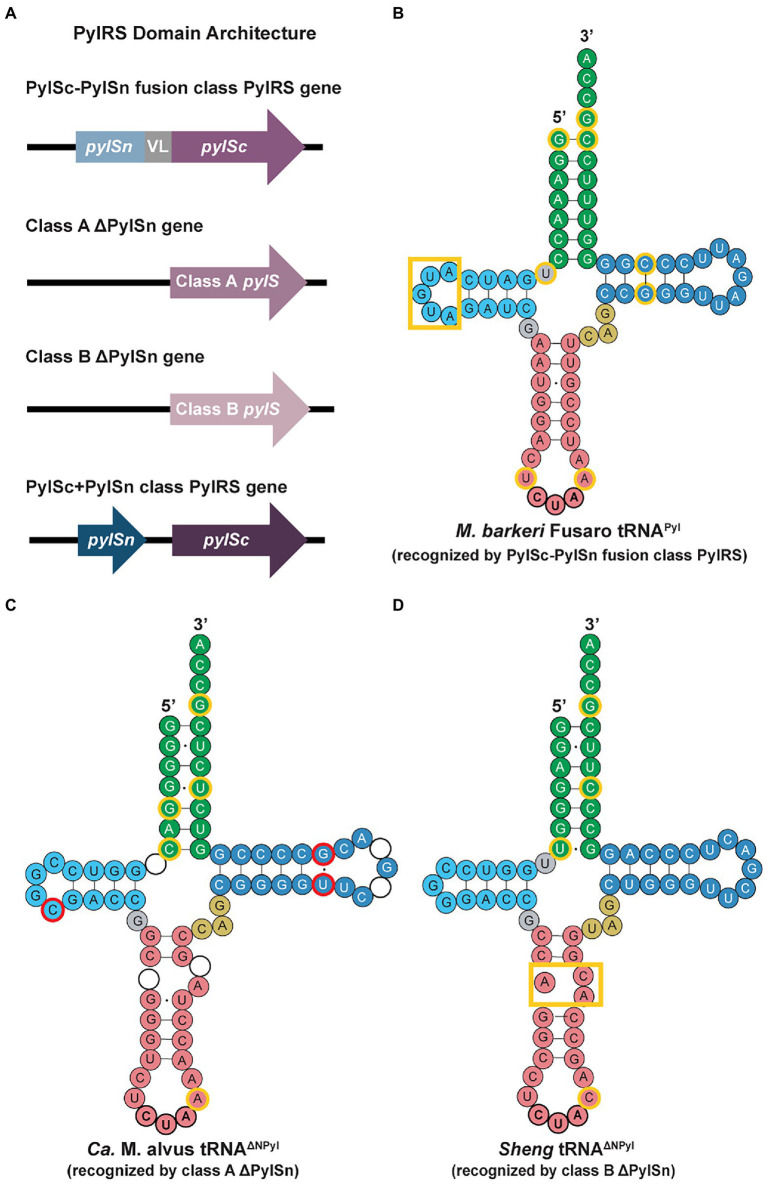
PylRS domain architectures and tRNA^Pyl^ secondary structures in archaea. **(A)** Domains of the PylRS genes from the different enzyme classes highlight the lack of an N-terminal domain for the class A and class B ΔNPylRS and the encoding of the CTD and NTD of PylRS in separate genes for the PylSc+PylSn class. tRNA^Pyl^ secondary structures recognized by **(B)** PylSc-PylSn fusion class PylRS, **(C)** class A ΔNPylRS, and **(D)** class B ΔNPylRS (Dunkelmann et al., 2020). The identity elements for all three classes of tRNA^Pyl^ molecules are outlined in yellow. The tRNA^Pyl^ recognized by class A and B ΔNPylRS (tRNA^ΔNPyl^) noticeably contain a break or bulge in the anticodon stem (pink). For the class A tRNA^ΔNPyl^, nucleotides highlighted in red represent bases that are missing in the sequence of other class A tRNA^ΔNPyl^, whereas circles without nucleotides represent those that are missing in the *Ca.* M. alvus sequence but present in others of the same class.

PylRS has a CTD with a structure similar to that of other class II aminoacyl-tRNA synthetases (aaRSs; Eriani et al., 1990). It is notable that the structure of PylRS resembles that of phenylalanyl-tRNA synthetase, as both enzymes belong to the same subclass within the class II aaRSs (Kavran et al., 2007). The CTD of PylRS contains the highly conserved catalytic core, which accommodates Pyl and ATP for aminoacylation (Kavran et al., 2007). Some classes of PylRS enzymes also include an N-terminal RNA-binding domain that does not resemble any currently known protein domains (Jiang and Krzycki, 2012; Wan et al., 2014). In archaea, PylRS is commonly encoded by a single *pylS* gene, whereas the CTD and NTD of all bacterial and certain archaeal PylRS are encoded by two separate genes, *pylSn* and *pylSc* (Yuan et al., 2010b; Guo et al., 2022). The PylRS enzymes found in archaea can be classified into three major classes: the PylSn-PylSc fusion class, the PylSn+PylSc class, and the ΔPylSn class (Figure 3A; Dunkelmann et al., 2020; Krahn et al., 2020; Guo et al., 2022).

#### PylSn-PylSc fusion class

Archaeal PylRS enzymes of the PylSn-PylSc fusion class, such as those from *Methanosarcinaceae*, have both a CTD and NTD, joined together by a linker. Although *in vitro* experiments suggest the NTD is dispensable, activity *in vivo* is not possible without the recruitment of tRNA^Pyl^ by the PylRS NTD (Herring et al., 2007; Nozawa et al., 2009; Suzuki et al., 2017).

Crystal structures revealed that the NTD of *Methanosarcina mazei* PylRS interacts with the T-loop and variable loop of tRNA^Pyl^, while the CTD interacts with the other side of tRNA^Pyl^ (Suzuki et al., 2017). This interaction distinguishes tRNA^Pyl^ with its small (three nucleotide) variable loop (Figure 3) from the other canonical tRNAs with four or more nucleotides. Among the PylRS enzymes of the PylSn-PylSc class, there is a wide range of sequence variability. In particular, the linker region varies greatly in length, from the *Methanococcoides burtonii* linker that is 14 amino acids in length, to the *Myceliophthora thermophila* linker of 72 amino acids (Herring et al., 2007). In addition, unlike the aaRSs for most canonical amino acids, there are no interactions between the anticodon of tRNA^Pyl^ and the CTD or NTD of PylRS, facilitating the application of PylRS for encoding non-canonical amino acids at different codons.

#### PylSn+PylSc class

Recently, seven archaeal genomes have been found to encode the NTD and CTD as two distinct genes, *pylSn* and *pylSc*, as commonly seen in bacteria (Figure 3A; Guo et al., 2022). Two examples are the PylRS enzymes found in the Methanomicrobia archaeon JdFR-19 and *Candidatus* Hydrothermarchaeum profundi (Guo et al., 2022). In these cases, the *pylSc* sequences of these two archaeal lineages more closely resemble bacterial *pylSc* sequences in *Acetohalobium arabaticum* and *Halarsenatibacter silvermanii*, rather than those in archaea. Aligning with previously discovered similarities between the Pyl operon in *A. arabaticum* and archaea, the similarities between the *pylSc* of these archaeal and bacterial lineages further support the hypothesis of LGT of the Pyl operon from archaea to bacteria (Borrel et al., 2014; Guo et al., 2022).

Though the PylRS of JdFR-19 belongs to the PylSc+PylSn class, this archaeon is a closely related relative of the *Methanosarcinaceae*, which encodes PylRS of the PylSc-PylSn fusion class (Guo et al., 2022). Another close relative to *Methanosarcinaceae* is the organism *Methermicoccus shengliensis*, which encodes the ΔPylSn class of PylRS (Cheng et al., 2007). In combination, these two observations suggest fusion of the PylSc and PylSn domains occurred in an ancestor of the *Methanosarcinaceae* (Guo et al., 2022). As this class of PylRS enzyme has only been recently discovered in archaea and mostly in uncultivated lineages, the sequences and structures of these enzymes still need to be further explored.

#### ΔPylSn functional classes A and B

In certain species of the *Methanomassiliicoccus* and *Candidatus* Sifarchaeia lineages, *pylS* genes are truncated by about 140 residues, resembling that of *pylSc* in bacteria (Borrel et al., 2014). Genomic searches revealed that there are no genes resembling bacterial *pylSn*, thus, these PylRS enzymes belong to the ΔPylSn class and have been named ΔNPylRS (Borrel et al., 2014; Willis and Chin, 2018). Despite the necessity of the NTD for recruitment and aminoacylation activity *in vivo*, enzymes from the ΔPylSn class demonstrate an equal or higher level of activity in comparison to those of the PylSc-PylSn fusion class (Willis and Chin, 2018; Dunkelmann et al., 2020). Additionally, there is strong sequence and structural alignment between the ΔPylSn PylRS and the CTD from the PylSc-PylSn fusion class, suggesting the catalytic ability of these enzymes in the absence of the NTD depends on the unique structure of their cognate tRNAs (Krahn et al., 2020). It has yet to be confirmed whether the PylSc-PylSn fusion class or the ΔPylSn class is the ancestral form of PylRS. However, given that a greater number of Pyl-encoding archaea and bacteria contain both the CTD and NTD of PylRS, it is likely that the ancestral PylRS included both the CTD and NTD, and the N-terminus of the *pylS* gene was then lost in the 7th order methanogens (Borrel et al., 2014).

Within the ΔPylSn class, there are two clusters of PylRS enzymes (class A and class B) distinguished by their sequences (Dunkelmann et al., 2020). Similar clustering is reflected in the cognate tRNA^Pyl^ based on characteristic differences. Some ΔPylSn class A enzymes are active with tRNA^ΔNPyl^ (tRNA^Pyl^ that is recognized by ΔNPylRS) from both classes with a preference for class A tRNA^ΔNPyl^, while others are naturally orthogonal (Dunkelmann et al., 2020). Class B enzymes, on the other hand, are only active with tRNA^ΔNPyl^ from the same class (Dunkelmann et al., 2020). This specificity suggests the amino acid sequences of class A and B ΔNPylRS evolved to recognize the class-specific identity elements of tRNA^ΔNPyl^. Furthermore, many ΔNPylRS are orthogonal to the cognate tRNA^Pyl^ of PylRS in the PylSc-PylSn fusion class (Dunkelmann et al., 2020). However, this orthogonality is unidirectional, as PylRS of the PylSc-PylSn fusion class are active with both class A and B tRNA^ΔNPyl^. To achieve full orthogonality, the variable loop of class A and B tRNA^ΔNPyl^ need to be expanded to avoid recognition by PylSc-PylSn fusion class PylRS (Suzuki et al., 2017; Dunkelmann et al., 2020). Taking advantage of the specificity of these ΔNPylRS enzymes for tRNA^ΔNPyl^ within the same class, they have become an important element in strategies for engineering orthogonality to insert multiple non-canonical amino acids in the same protein.

### tRNA^Pyl^ structure

All characterized tRNA^Pyl^ have a well-conserved structure consisting of some distinguishing features: a three-base variable loop, a small D-loop of 3–5 nucleotides, and a long anticodon stem of 6–8 bases in length (Tharp et al., 2018; Figure 3). These tRNA^Pyl^ also contain the G73 discriminator base, recognized by PylRS. Given the recent discovery of archaea encoding PylSc+PylSn class PylRS, the sequences and structures of the tRNA^Pyl^ isoacceptors for this class of PylRS enzymes has yet to be explored (Guo et al., 2022). Thus, our discussion of tRNA^Pyl^ structures below will be limited to those recognized by the PylSc-PylSn fusion class and the ΔPylSn class of archaeal PylRS, for which there are additional distinguishing traits which separate their respective tRNA^Pyl^. Despite these differences, it is still unknown whether the differences between tRNA^ΔNPyl^ and other tRNA^Pyl^ molecules is a result of adaptation to the loss of the NTD of PylRS, or whether mutations producing tRNA^ΔNPyl^ promoted the loss of the NTD (Borrel et al., 2014; Willis and Chin, 2018).

#### tRNA^Pyl^ recognized by PylSc-PylSn fusion enzymes

The tRNA^Pyl^ sequence of the PylSc-PylSn class is quite divergent; however, there are distinct secondary structure features that are conserved and different from tRNA^Pyl^ from other PylRS classes. These unique structural elements include a D-loop of 5 bases and a single base separating the D-stem from the acceptor stem, an identity element in bacterial tRNA^Pyl^ (Gaston et al., 2011a; Figure 3B). The small D-loop of tRNA^Pyl^ is essential for forming the compact core, which is recognized by tRNA binding domain 1, the C-terminal tail, and the α6 helix of the opposing protomer that forms the core binding surface of PylRS (Nozawa et al., 2009). Sequence-specific recognition of tRNA^Pyl^ by PylRS is regulated by the presence of the U33 and A37 nucleotides flanking the anticodon, the G53:C63 T-stem base pair, and the G1:C72 acceptor stem base pair (Ambrogelly et al., 2007).

#### tRNA^Pyl^ recognized by class A and B ΔNPylRS enzymes

For tRNA^ΔNPyl^ of the 7th order methanogens, certain members of this archaeal lineage encode for multiple *pylT* genes with different sequences within the genome (Borrel et al., 2014). The sequences of tRNA^ΔNPyl^ also vary greatly (Figures 3C,D). In comparison to tRNA^Pyl^ recognized by the PylSc-PylSn class, the D-loop of tRNA^ΔNPyl^ is shortened to only 3 or 4 bases. The D-stem and acceptor stem may be separated by up to 2 bases, and the conserved variable loop sequence CAG matches that of tRNA^Pyl^ found in the bacterial species *Desulfitobacterium hafniense*. However, the most notable feature of tRNA^ΔNPyl^ is the broken anticodon stem (Figures 3C,D). This is characteristic of tRNA^ΔNPyl^ from all 7th order methanogens, although the shape of the small loop formed by this break varies between species. The tRNA^ΔNPyl^ molecules recognized by class A ΔNPylRS contain A37 adjacent to the CUA anticodon (Figure 3C), like in tRNA^Pyl^ of *Methanosarcinaceae*, whereas the tRNA^ΔNPyl^ molecules recognized by class B ΔNPylRS contain C37 (Dunkelmann et al., 2020; Figure 3D). The C37A mutation in the class B tRNA^ΔNPyl^ molecules leads to higher levels of amber codon suppression by the class B ΔNPylRS/tRNA^ΔNPyl^ pair (Figure 3D). Other differences in the sequence of class A and class B tRNA^ΔNPyl^ molecules are found in the acceptor stem, T-stem, and T-loop. Sequence diversity also exists within the class A and B tRNA^ΔNPyl^, but the secondary structure of the tRNA^ΔNPyl^ molecules is conserved within each class (Figures 3C,D).

### Regulation of Pyl insertion

In contrast to the strict sequence constraints for Sec insertion, Pyl insertion is likely regulated by competition between Pyl-tRNA^Pyl^ and an archaeal release factor at ambiguous UAG codons, thus requiring no additional proteins or mRNA motifs for Pyl-specific insertion (Zhang et al., 2005). Following this model of competition, if early termination occurs at a Pyl-encoded UAG codon, the truncated proteins are degraded (Alkalaeva et al., 2009). Conversely, unintended read-through of UAG codons with Pyl is controlled by termination at a UAA or UGA codon located closely downstream (Zhang et al., 2005). Analysis of Pyl-encoding genomes has revealed a stem-loop structure similar to that of the SECIS element, termed the PYLIS element, immediately downstream of UAG codons in certain Pyl-encoding gene sequences (Hemmerle et al., 2020). However, the PYLIS element is not present in all genes that code for Pyl, nor is it necessary for Pyl insertion (Alkalaeva et al., 2009). There is also little sequence and structure conservation between the PYLIS element of different species (Ambrogelly et al., 2007).

The Pyl system is highly concentrated in a limited number of bacterial species and in members of the methanogenic archaeal phyla *Halobacteriota* and *Thermoplasmatota*, which are sister groups (Zhang et al., 2022). Thus, the current state of the field supports the idea that Pyl was added to the genetic code of the common ancestor of *Halobacteriota* and *Thermoplasmatota* to better survive in extreme environments, only to be lost later in evolutionary history (Brugère et al., 2018). In particular, many Pyl-encoding archaeal species are methylotrophic methanogens that inhabit anoxic environments. In this case, possessing Pyl machinery is necessary to produce methylamine methyltransferase, an enzyme which generates methylamines for methanogenesis (Ambrogelly et al., 2007). Subsequently, the Pyl system may have been introduced into bacteria through lateral gene transfer (LGT) from the 7th order methanogens, as the *Methanomassiliicoccus* lineage has closer evolutionary relations to bacteria than the *Methanosarcinaceae* (Borrel et al., 2014). In this case, LGT would have occurred prior to the loss of the NTD in the 7th order methanogens. Further discussion of the Pyl system in archaea can be found in previous reviews (Krzycki, 2013; Wan et al., 2014; Baumann et al., 2018; Brugère et al., 2018; Tharp et al., 2018).

## tRNA-dependent amino acid biosynthesis

**Figure 4:**
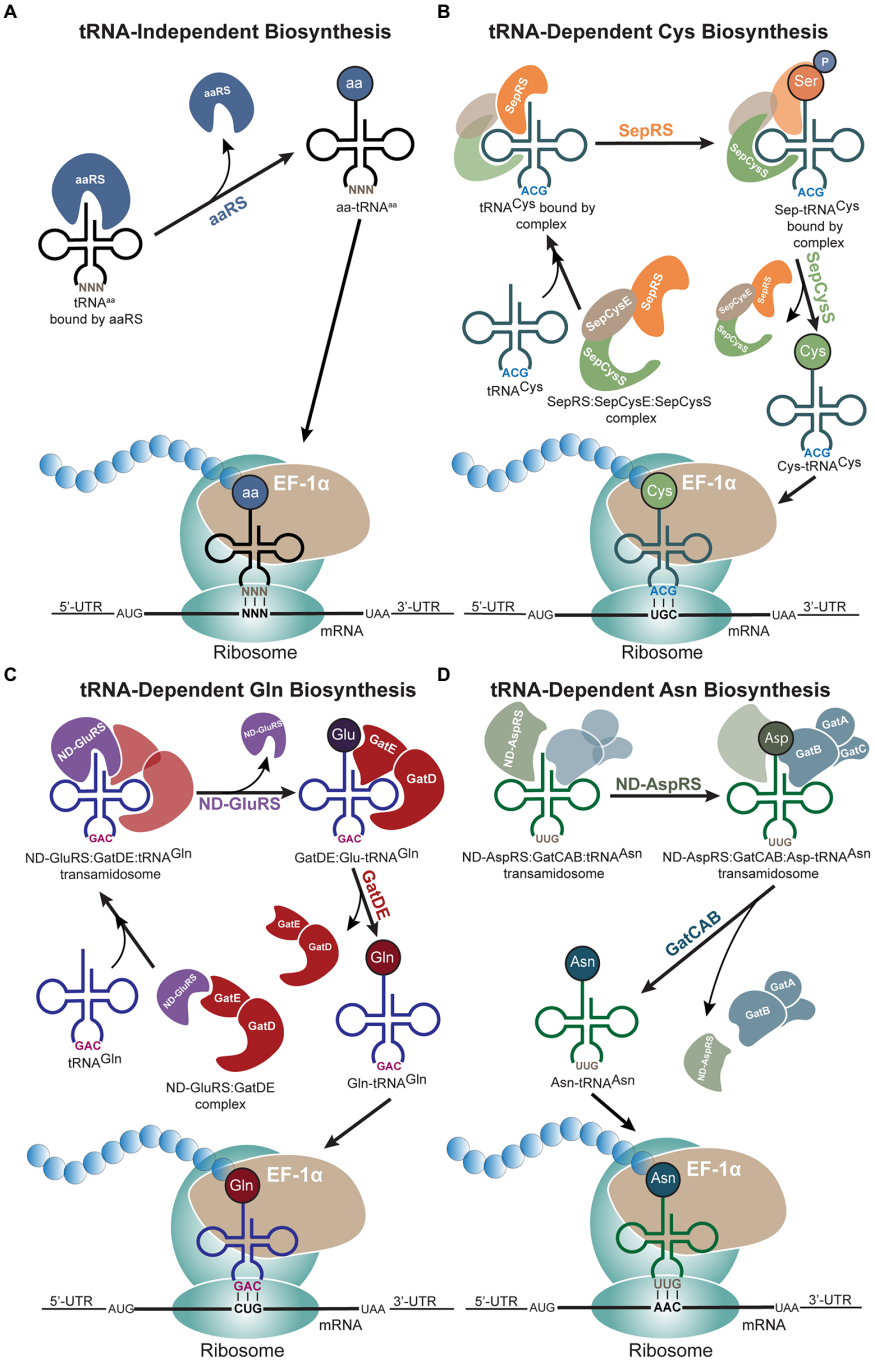
tRNA-dependent biosynthesis of amino acids in archaea. **(A)** Schematic of the traditional tRNA-independent biosynthesis of amino acids, **(B)** cysteine (Cys), **(C)** glutamine (Gln), and **(D)** asparagine (Asn) proceed through different mechanisms. **(B)** Protein complex formation precedes binding of tRNA^Cys^. The two-step process of Cys biosynthesis occurs through *O*-phosphoseryl-tRNA synthetase (SepRS) and SepCysS, while the protein-tRNA^Cys^ complex remains intact until phosphoseryl is converted to Cys. **(C)** tRNA-dependent Gln biosynthesis also begins with protein complex formation followed by binding of tRNA^Gln^. After non-discriminating glutamyl-tRNA synthetase (ND-GluRS) aminoacylates tRNA^Gln^ with glutamic acid (Glu), ND-GluRS dissociates for the GatDE heterodimer to catalyze the conversion of Glu to Gln. **(D)** Non-discriminating aspartyl-tRNA synthetase (ND-AspRS) and GatCAB form a complex through binding tRNA^Asn^. This complex remains intact even after ND-AspRS aminoacylates tRNA^Asn^ with aspartic acid (Asp), and the complex only dissociates once GatCAB converts Asp to Asn. In all pathways, archaeal EF-1α brings the aminoacyl-tRNAs (aa-tRNAs) to the ribosome.

Faithful translation of a protein gene sequence depends on the successful creation of aminoacyl-tRNAs (aa-tRNAs) through correct aminoacylation of an amino acid onto its cognate tRNA. The most direct path for aa-tRNA production is through aminoacylation of a cognate tRNA by its designated aaRS (Figure 4A). However, archaea do not encode specific aaRSs for the full set of 20 canonical amino acids, thus resulting in indirect creation of aa-tRNAs through tRNA-dependent amino acid biosynthesis (Wilcox and Nirenberg, 1968). Similar to Sec biosynthesis, a specific tRNA isoacceptor is misacylated by a non-cognate aaRS, and the misacylated aa-tRNA is converted to the desired aa-tRNA through tRNA-dependent enzymes (Sheppard et al., 2008b). In archaea, three of the 20 canonical amino acids can be synthesized in this tRNA-dependent manner: cysteine (Cys), glutamine (Gln), and asparagine (Asn; Figures 4B–D). For all three amino acids, tRNA-dependent biosynthesis progresses through the formation of a complex—the transsulfursome for Cys and the transamidosome for Gln and Asn (Mukai et al., 2021). The formation of a complex in these cases prevents the misacylated aa-tRNA from existing freely inside the cell and being recognized by the elongation factor, protecting against misincorporation, while also efficiently shuttling the intermediate between the enzymes. The similarities between these indirect biosynthesis routes with that of Sec biosynthesis suggest the possibility of a PSTK:SepSecS:tRNA^Sec^ complex (Mukai et al., 2021). However, as tRNA^Sec^ is recognized by EFSec rather than the general elongation factor, the Sec biosynthesis pathway prevents mistranslation even without complex formation, warranting further investigation of the Sec biosynthesis pathway.

### Cysteine biosynthesis

In most organisms, translation of Cys occurs through the canonical path involving aminoacylation of Cys-specific tRNA isoacceptor (tRNA^Cys^) by cysteinyl-tRNA synthetase (CysRS). However, certain bacteria, class I methanogens, and Asgardarchaeota either lack or do not require the gene encoding CysRS (Sauerwald et al., 2005; Mukai et al., 2021). In these archaeal lineages, Cys is biosynthesized on tRNA^Cys^, forming Cys-tRNA^Cys^ through a pathway similar to that of Sec biosynthesis (Figures 1, 4B). First, tRNA^Cys^ is acylated with Sep by *O*-phosphoseryl-tRNA synthetase (SepRS) to produce Sep-tRNA^Cys^, which is then converted by Sep-tRNA:Cys-tRNA synthase (SepCysS) to Cys-tRNA^Cys^ (Figure 4B). For incorporation into proteins, Cys-tRNA^Cys^ is delivered to the ribosome by the archaeal elongation factor 1 alpha (EF-1α) and inserted at Cys codons.

SepRS exists as an α_4_ tetramer with each SepRS monomer containing an N-terminal extension, a catalytic domain, an insertion domain, and a C-terminal anticodon-binding domain (Fukunaga and Yokoyama, 2007). SepRS is specific for the amino acid Sep and the GCA anticodon. Methylated G37, the G1-C72 base pair, and the U73 discriminator base serve as identity elements on tRNA^Cys^ for SepRS binding (Fukunaga and Yokoyama, 2007). SepRS is designed to also prevent EF-1α from binding Sep-tRNA^Cys^ and misincorporating Sep at Cys codons. This is accomplished through the SepRS insertion domain blocking the EF-1α binding site. In addition, gel filtration chromatography experiments have demonstrated that SepRS, SepCysS, and SepCysE form the SepRS:SepCysE:SepCysS complex in class I methanogens (Liu et al., 2014; Mukai et al., 2017). It has been proposed that SepCysE first facilitates an interaction between SepRS and SepCysS to form a complex for recognition of tRNA^Cys^ (Liu et al., 2014). Once tRNA^Cys^ is bound by the SepRS:SepCysE:SepCysS complex, the CCA tail of tRNA^Cys^ is aminoacylated with Sep by SepRS, then transferred to the catalytic site of SepCysS for Sep to Cys conversion (Liu et al., 2014). The SepRS:SepCysE:SepCysS complex releases Cys-tRNA^Cys^ once the conversion is complete (Liu et al., 2014; Figure 4B). However, the mechanism of tRNA-dependent Cys biosynthesis differs slightly in Asgardarchaeota, as their SepCysE proteins lack the N-terminal helix needed for binding of SepRS (Mukai et al., 2021). This suggests that SepRS is separate from the SepCysS:SepCysE complex in these organisms.

SepCysS binding occurs through the recognition of U73 on tRNA^Cys^, while the CTD of SepCysE binds tRNA^Cys^ in a non-specific manner (Chen et al., 2017). Through the formation of this complex, Sep-tRNA^Cys^ bypasses release into solution and the risk of being bound by EF-1α. Homologs of the NTD of SepCysE also exist in other archaeal genomes, either as an additional domain of SepCysS or as a split gene upstream of the SepCysS gene, which may suggest LGT events between the class I, II, and III methanogens and *Lokiarchaeota* (Mukai et al., 2017). Though no archaea have been found to encode two copies of SepRS, which has high specificity for tRNA^Cys^, some archaea contain two copies of the more ambiguous SepCysS gene (Mukai et al., 2017). These two SepCysS gene copies can belong to the same clade or to different clades, but both genes produce SepCysS enzymes that serve the same function (Mukai et al., 2017). The duplication of SepCysS may enhance tRNA-dependent Cys biosynthesis under stressful environmental conditions.

Although not all methanogens lack CysRS and require the tRNA-dependent Cys biosynthesis pathway, SepRS and SepCysS are conserved in all methanogenic species, except *Methanobrevibacter* and *Methanosphaera* (Liu et al., 2014). Structural analysis also supports that SepRS and SepCysS evolved prior to the tRNA-independent Cys biosynthesis pathway (Zhang et al., 2012). Thus, the tRNA-dependent strategy is believed to be the ancestral mode of Cys biosynthesis. However, once tRNA-independent Cys biosynthesis evolved in bacteria, LGT introduced bacterial CysRS into class II and III methanogens, causing them to lose SepCysE, which is only found in class I methanogens (O'Donoghue et al., 2005; Fukunaga and Yokoyama, 2007; Liu et al., 2014). Since Sep-tRNA^Cys^ is vulnerable to hydrolysis in higher temperatures, it follows that an accessory protein would be advantageous for maintaining Cys-tRNA^Cys^ biosynthesis, leading SepCysE to coevolve with SepRS and SepCysS to overcome the extreme conditions inhabited by class I methanogens (Liu et al., 2014).

### Glutamine biosynthesis

All known archaeal and most bacterial lineages lack the gene encoding glutaminyl-tRNA synthetase (GlnRS). Thus, Gln is biosynthesized on its tRNA isoacceptor, tRNA^Gln^, in a two-step process as observed with Cys biosynthesis (Sheppard et al., 2008b; Figures 4B,C). tRNA^Gln^ is first aminoacylated with glutamic acid (Glu) by the non-discriminating glutamyl-tRNA synthetase (ND-GluRS) to produce Glu-tRNA^Gln^ (Figure 4C). At this point, Glu-tRNA^Gln^ is converted to Gln-tRNA^Gln^ through glutamyl-tRNA^Gln^ amidotransferase (Glu-AdT; Wilcox and Nirenberg, 1968; Lapointe et al., 1986; Schön et al., 1988). In archaea, the heterodimer GatDE serves the role of Glu-AdT (Tumbula et al., 2000). During biosynthesis, ND-GluRS and GatDE form a tRNA-independent complex, the archaeal-specific transamidosome (ND-GluRS:GatDE), forming interactions on surfaces not required to bind the tRNA (Rampias et al., 2010). GatDE increases the affinity of ND-GluRS for tRNA^Gln^ by initiating the reaction needed to discriminate between tRNA^Gln^ and tRNA^Glu^ through interaction of GatDE with the D-loop and the A1:U72 base pair of tRNA^Gln^ (Oshikane et al., 2006; Rampias et al., 2010). Once the transamidosome binds tRNA^Gln^, forming ND-GluRS:GatDE:tRNA^Gln^, the ND-GluRS aminoacylates the 3′-end of tRNA^Gln^ (Figure 4C). After aminoacylation, the ND-GluRS unbinds, resulting in GatDE:Glu-tRNA^Gln^, and a conformational change occurs to flip the glutamylated end of tRNA^Gln^ into the catalytic site of GatE for transamidation of Glu to Gln (Rampias et al., 2010). ND-GluRS must unbind after aminoacylation since both ND-GluRS and GatDE bind the minor groove of the tRNA acceptor stem, thus ND-GluRS blocks the 3′-end of the tRNA from accessing the catalytic pocket of GatDE (Rampias et al., 2010). It has also been proposed that Gln-tRNA^Gln^ may be biosynthesized without the formation of a transamidosome, though this method of Gln biosynthesis is less efficient due to the reduced ability of ND-GluRS to discriminate against tRNA^Glu^ (Rampias et al., 2010). In this case, ND-GluRS aminoacylates tRNA^Gln^ and releases Glu-tRNA^Gln^. The free Glu-tRNA^Gln^ then undergoes transamidation by GatDE (Rampias et al., 2010).

The absence of a gene encoding GlnRS in all archaeal species is an anomaly given the common phenomenon of LGT for aaRS genes between the three domains (Woese et al., 2000). This is hypothesized to be a result of the many differences between tRNA^Gln^ preventing recognition by the eukaryotic and bacterial GlnRS (Tumbula et al., 2000). In fact, archaeal tRNA^Gln^ differs from bacterial tRNA^Gln^ at over half of the identity elements. Notably, archaeal tRNA^Gln^ contains the A73 discriminator base, whereas bacterial tRNA^Gln^ contains the G73 discriminator base and eukaryotic tRNA^Gln^ uses U73 (Marck and Grosjean, 2002). In contrast to the highly conserved G1:C72 base pair and G12:C23 base pair in eukaryotic tRNA^Gln^, archaeal tRNA^Gln^ possess A1:U72 and C12:G23 (Marck and Grosjean, 2002; Mallick et al., 2005). An alternate explanation for the unique identity elements of archaeal tRNA^Gln^ is for adaption to the tRNA-dependent Gln biosynthesis route.

### Asparagine biosynthesis

The majority of archaea and bacteria do not encode asparaginyl-tRNA synthetase (AsnRS), thus these archaeal and bacterial lineages also biosynthesize Asn through transamidation and the formation of a transamidosome, in a process that parallels that of tRNA-dependent Gln biosynthesis (Sheppard et al., 2008b; Figures 4C,D). Non-discriminating aspartyl-tRNA synthetase (ND-AspRS) first aminoacylates asparaginyl-tRNA (tRNA^Asn^) with aspartic acid (Asp), and Asp-tRNA^Asn^ amidotransferase (Asp-AdT) converts Asp-tRNA^Asn^ to Asn-tRNA^Asn^ (Curnow et al., 1996; Becker et al., 1997). In archaea, the heterotrimeric enzyme GatCAB acts specifically as the Asp-AdT, while GatCAB functions as both the Glu-AdT and Asp-AdT in bacteria (Sheppard et al., 2008a,b). Additionally, the structure of the archaeal and bacterial Asn-transamidosomes differ given the absence of the GAD domain in the archaeal transamidosome, resulting in slower release of Asn-tRNA^Asn^ (Suzuki et al., 2015). In contrast to Gln biosynthesis, the transamidosome formed for Asn biosynthesis (ND-AspRS:tRNA^Asn^:GatCAB) is dependent on tRNA^Asn^ binding and is stable throughout the sequential reactions (Bailly et al., 2007). This complex functions to protect Asn-tRNA^Asn^ from deacylation and prevent Asp-tRNA^Asn^ from being recognized by archaeal EF-1α as the intermediate is kept within the transamidosome until Asn-tRNA^Asn^ is produced (Bailly et al., 2007; Huot et al., 2007; Liu et al., 2014). Differences between the two transamidosomes may be due to their recognition sites. Both ND-GluRS and Glu-AdT bind the minor groove and therefore cannot both interact with the tRNA at the same time. However, ND-AspRS binds the tRNA major groove, opposite to GatCAB, relying on the tRNA to make contact (Arnez and Moras, 1997; Bailly et al., 2007).

Phylogenetic analysis of tRNA^Asn^ shows that division between tRNA^Asn^ sequences of archaea, bacteria, and eukaryotes are not very rigid (Sheppard and Söll, 2008). Additionally, archaeal tRNA^Asn^ possesses the G73 discriminator base and the GUU anticodon, which are identity elements needed for recognition by bacterial AsnRS (Giegé et al., 1998; Sheppard and Söll, 2008). The presence of these identity elements supports the possibility of LGT, resulting in certain archaeal species encoding for AsnRS and losing the tRNA-dependent Asn biosynthesis system.

For the indirect biosynthesis of Gln and Asn, comparative phylogenetic analysis suggests both GatDE and GatCAB were present in LUCA, despite GatDE now only being found in archaea (Sheppard and Söll, 2008). The specificity of archaeal GatDE for bases on archaeal Glu-tRNA^Gln^ suggests the two co-evolved, resulting in a great divergence of archaeal tRNA^Gln^ from its bacterial and eukaryotic homologs and the lack of LGT of GlnRS to archaea. In contrast, archaeal GatCAB evolved selection against Glu-tRNA^Gln^ instead of recognition of Asp-tRNA^Asn^, leading to conserved characteristics between tRNA^Asn^ of all three domains and the potential for LGT of AsnRS to archaea (Namgoong et al., 2007). It is also possible that subpopulations of LUCA encoded only GatCAB while others encoded for both GatCAB and GatDE. In this situation, bacteria evolved from the subpopulation containing only GatCAB, whereas archaea evolved from those that possessed both AdTs (Sheppard and Söll, 2008).

## Multiple genetic coding systems within one organism

Archaea are the first domain of life to be found containing multiple genetic code systems (Mukai et al., 2017). As many archaeal species inhabit extreme conditions, this is their strategy to survive and overcome various environmental challenges, including heat, high salt concentrations, and anoxic conditions (Reed et al., 2013). Within these archaea, the tRNA isoacceptors and enzymes associated with amino acid biosynthesis or aminoacylation are often unique in structure and sequence from those of the canonical archaeal tRNA and aaRSs (Mukai et al., 2017; Sun et al., 2021; Zhang et al., 2022). As many of these archaeal lineages belong to recently discovered or characterized phylogenetic groups (Mukai et al., 2017; Sun et al., 2021; Zhang et al., 2022), further study of these archaea and the different coding systems they possess will shed light on the evolutionary history of different amino acids of the genetic code and their biosynthesis strategies.

### Co-occurrence of Sec and Pyl coding systems

The recently discovered Asgardarchaeota lineage, *Candidatus* Sifarchaeia, is the first identified non-methanogenic archaea to encode all the necessary *pyl* genes (Sun et al., 2021). This is likely due to Sifarchaeia encoding a Pyl-containing methylamine methyltransferase involved in a methanogen-like pathway, which is not seen in other Asgardarchaeota genomes. The organization of the *pyl* genes in Sifarchaeia is also unique, as *pylTS* are encoded in a separate cluster from *pylBCD*, differing from the uninterrupted *pylTSBCD* cluster seen in most archaeal genomes (Gaston et al., 2011a). However, the tRNA^Pyl^ encoded by Sifarchaeia is unusual as it contains a GC tail (Sun et al., 2021), discriminating it from archaeal tRNA^Pyl^ homologs that contain a CCA tail (Tharp et al., 2018). Furthermore, Sifarchaeia have a significantly higher usage of the UAG codon as a genuine termination signal in comparison to other Pyl-encoding archaea (Zhang et al., 2005). The high usage of the UAG stop codon introduces the question of how Sifarchaeia differentiates between insertion of Pyl or termination, as there have yet to be any signals or factors discovered to regulate Pyl insertion in the way Sec insertion is regulated (Sun et al., 2021). A hypothesis is that Pyl biosynthesis may only become active in the presence of methylamines.

**Figure 5:**
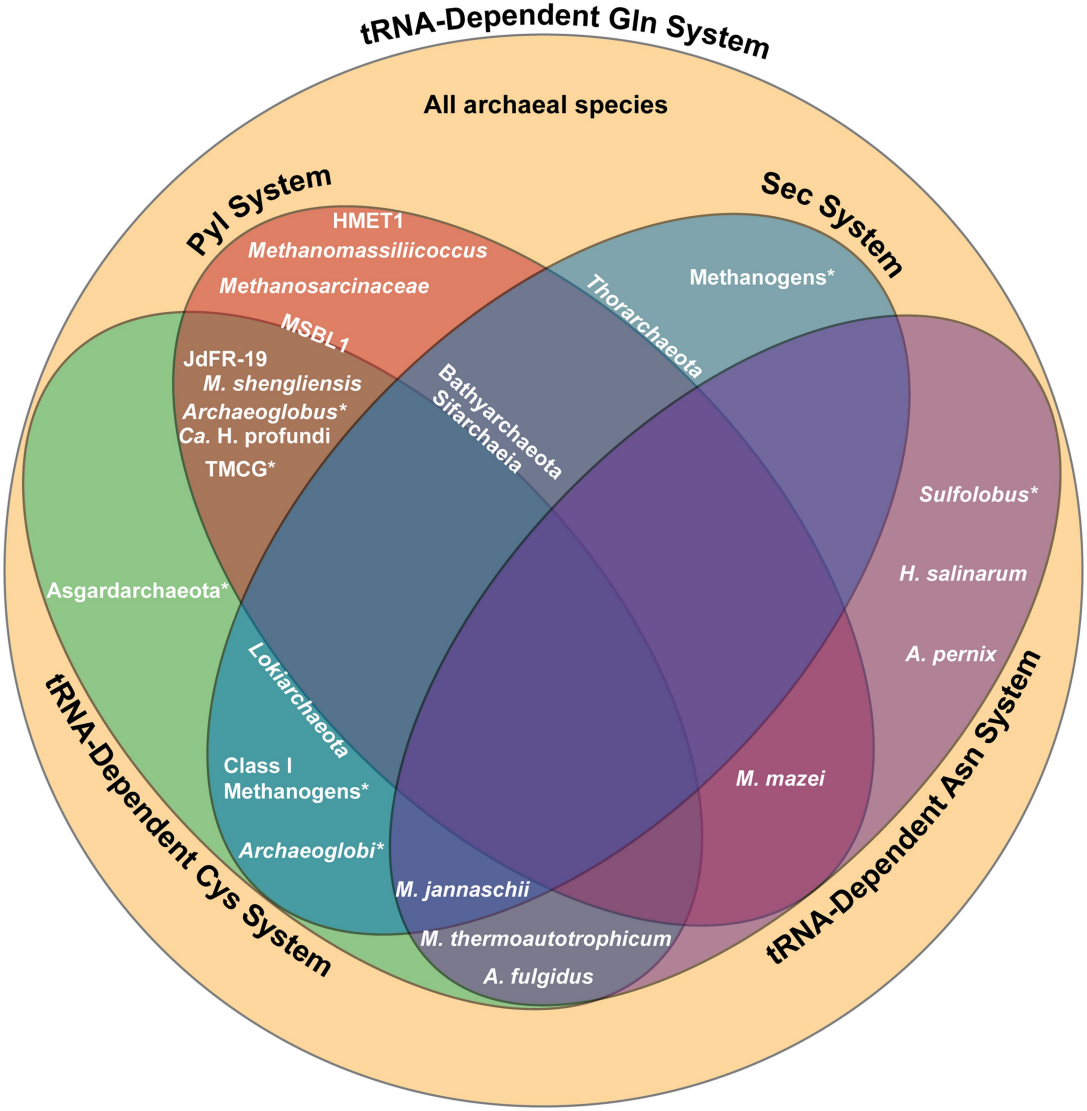
Summary of the genetic code systems found in various archaeal lineages. Lineages positioned within a section possess the complete machinery for the designated system, while those located on the border of a section possess only partial machinery. An asterisk (*) indicates that only certain species within the lineage utilize the specified system. All archaea utilize the tRNA-dependent Gln biosynthesis system. Gln, glutamine; Sec, selenocysteine; Pyl, pyrrolysine; Cys, cysteine; Asn, asparagine; TMCG, Terrestrial Miscellaneous Crenarchaeota group; HMET1, *Candidatus* Methanohalarchaeum thermophilum HMET1; JdFR-19, Methanomicrobia archaeon JdFR-19; MSBL1, Mediterranean Sea Brine Lakes 1 archaeon SCGC-AAA382A20.

Besides the complete Pyl machinery, the canonical Sec biosynthesis machinery is also present in Sifarchaeia to encode three selenoproteins (Figure 5; Sun et al., 2021). Notably, tRNA^Sec^ in these organisms have a 6 bp D-stem, a common characteristic of eukaryotic and bacterial tRNA^Sec^, as well as other insertions and deletions that distinguish tRNA^Sec^ in Sifarchaeia from canonical archaeal tRNA^Sec^ (Santesmasses et al., 2017). *Lokiarchaeota*, which is found in the same phylum, also encode these same three selenoproteins, though it contains the canonical 7 bp D-stem of archaeal tRNA^Sec^ (Mariotti et al., 2016; Sun et al., 2021).

While Sifarchaeia is the only lineage within Asgardarchaeota that possesses complete Pyl machinery, *pylT* is found in other Asgardarchaeota, including the *Lokiarchaeota* and *Thorarchaeota* phyla (Figure 5; Sun et al., 2021). Current research suggests that the Sec and Pyl systems coexisted in the last Asgardarchaeota common ancestor, with Pyl recoding lost in other Asgardarcheota lineages through evolution and Sec recoding maintained. The only other known lineages to encode both Sec and Pyl is the bacterial lineage Desulfobacterota and the methylotrophic methanogen *Ca.* Bathyarchaeota (Borrel et al., 2014; Guo et al., 2022). All three lineages are found in anoxic marine sediments. The existence of these three lineages in such an environment suggests the synthesis of selenoproteins may have yielded benefits to surviving the harsh conditions through energy conservation and protection from oxidative stress (Sun et al., 2021).

### Co-occurrence of Pyl and tRNA-dependent Cys biosynthesis

Certain archaea, such as strains of the methanogenic *M. shengliensis* that use trimethylamines as precursors to methane, possess both the Pyl and the tRNA-dependent Cys biosynthesis systems (Figure 5; Mukai et al., 2017). The coexistence of the Pyl and indirect Cys biosynthesis systems may be due to the fact that Pyl-containing enzymes used in methylamine metabolism require iron–sulfur (Fe-S) proteins for anaerobic methanogenesis (Rother and Krzycki, 2010). In this case, the usage of SepCysS, an Fe-S protein, may provide an additional advantage to Pyl-containing enzymes over CysRS, which is not an Fe-S protein (Liu et al., 2016).

Archaeal lineages that make use of the Pyl and tRNA-dependent Cys biosynthesis systems mostly live in anoxic conditions, another extreme environment. For example, PylRS and SepRS co-occur in certain subgroups of *Archaeoglobus* and methanogens in the Terrestrial Miscellaneous Crenarchaeota group (TMCG), found in hot spring metagenomes (Figure 5; Mukai et al., 2017). In this case, archaea of the TMCG might favor the indirect Cys biosynthesis pathway due to the stability of Sep at high temperatures (Makino et al., 2016). Analyzing the metagenome of a deep marine ecosystem has also revealed three additional archaeal species that possess SepRS and PylRS (Mukai et al., 2017). In this case, phylogenetic analysis of these three archaeal species sheds light on the evolutionary process behind the distribution of the Pyl coding system in different domains. The sequences for the PylRS of these three species divide the bacterial PylRS clade, supporting the idea for LGT of the Pyl system between bacteria and archaea.

### Co-occurrence of Sec and tRNA-dependent Cys biosynthesis

Certain class I methanogens, including *M. jannaschii*, encode both the Sec and tRNA-dependent Cys biosynthesis systems (Figure 5). Moreover, a few Sec-encoding archaea outside of the class I methanogens also produce SepCysE, including *Lokiarchaeota* (Figure 5; Mukai et al., 2017). The presence of both these genetic code systems in these two archaeal groups suggests emergence of archaea prior to divergence of class I methanogens and *Lokiarchaeota*. Though SepCysS has specificity for the U73 determinant of tRNA^Cys^, it is also found to bind Sep-tRNA^Sec^ with a G73 in the absence of SepCysE (Yuan et al., 2010a). One *Archaeoglobi* species with components for tRNA-dependent Cys biosynthesis (SepRS and SepCysS) has a divergent Sec-encoding system which has a system to prevent tRNA ambiguity (Figure 5). This *Archaeoglobi* species has PSTK and SelB fused in a single open reading frame, similar to what is found in *Ca.* Bathyarchaeota, which also contain all machinery for tRNA-dependent Cys biosynthesis except for the SepCysE (Figure 5; Mukai et al., 2017). In the absence of SepCysE, this PSTK-SelB fusion is suggested to prevent unintended recognition of Sep-tRNA^Sec^ by SepCysS. This hypothesis does not hold for the Sifarchaeia lineage, also encoding SepRS and SepCysS, but lacking SepCysE (Figure 5; Sun et al., 2021). In this lineage, PSTK and SelB are not fused together, suggesting an alternate role and potential overlap between the Sec and Cys systems. The possible Sec/Cys cross-talk in Sifarchaeia is similar to how bacteria and eukaryotes are able to misincorporate Cys and Sec. This suggests that the eukaryotic Sec-encoding system may have evolved from the Sifarchaeia lineage (Turanov et al., 2009; Xu et al., 2010).

### Co-occurrence of two distinct PylRS and tRNA^Pyl^ pairs

Though most archaea only encode for a single PylRS and tRNA^Pyl^ pair, two distinct and mutually orthogonal PylRS/tRNA^Pyl^ pairs have been identified to coexist in the methanogenic species *M. luminyensis* B10 and the extremely halophilic euryarchaeal methanogen *Candidatus* Methanohalarchaeum thermophilum HMET1 (Figure 5; Zhang et al., 2022). Phylogenetic reconstruction suggests the duplication of *pylS* and *pylT* genes occurred independently in the two species. In addition, the Pyl biosynthesis genes *pylB*, *pylC*, and *pylD* are also present twice in the *M. luminyensis* B10 genome, whereas only *pylB* was duplicated in HMET1.

In depth analysis of the PylRS systems in HMET1 revealed their mutual orthogonality (Zhang et al., 2022). Both enzymes belong to the ΔPylSn class and share 53% of their sequences. While predicted to adopt similar 3D structures, the motif 2 loop of PylRS2 is shorter by a single amino acid compared to PylRS1. This minor difference is sufficient to discriminate between the two tRNA^Pyl^. PylRS1 with the complete motif 2 loop sequence recognizes tRNA^Pyl^1, the tRNA^Pyl^ isoacceptor containing the G73 discriminator base (*pylTG*), while PylRS2 recognizes tRNA^Pyl^2, the tRNA^Pyl^ with the non-canonical A73 discriminator base (*pylTA*; Guo et al., 2022). Moreover, there are additional base substitutions in the variable loop of *pylTA* tRNA which contribute to their mutual orthogonality (Zhang et al., 2022). From current research, the implications of the coexistence of these two orthogonal PylRS systems in HMET1 have yet to be determined, though it is suggested that they may each be under different environmental control or have different amino acid specificity.

Found in the same hypersaline environment as HMET 1, two copies of PylRS have also been found in the Mediterranean Sea Brine Lakes 1 archaeon SCGC-AAA382A20 (MSBL1) lineage (Figure 5; Guo et al., 2022). However, only *pylTA* was found in the MSBL1 genome while *pylTG* is absent (Guo et al., 2022). Additionally, the gene encoding SepRS has been found in MSBL1, though no other components of tRNA-dependent Cys biosynthesis have been identified (Figure 5; Mukai et al., 2017). As MSBL1 is an uncultured archaeal lineage, its genome is still currently incomplete (Mwirichia et al., 2016), thus further characterization of the MSBL1 genome may reveal the missing components of these genetic code systems.

### Co-occurrence of multiple tRNA-dependent amino acid biosynthesis systems

As all known archaea biosynthesize Gln indirectly and many also biosynthesize Asn indirectly, the co-occurrence of multiple tRNA-dependent amino acid biosynthesis pathways is common. Archaeal species that are known to lack the gene encoding AsnRS and perform both tRNA-dependent Gln and Asn biosynthesis include *Aeropyrum pernix*, *Archaeoglobus fulgidus*, *Halobacterium salinarum*, *Methanothermobacter thermoautotrophicum*, *M. jannaschii*, *M. mazei*, and two *Sulfolobus* species (Figure 5; Tumbula et al., 2000). Furthermore, the species *M. thermoautotrophicum* and *A. fulgidus* also contain machinery for tRNA-dependent Cys biosynthesis, and *M. jannaschii* contains both Sec and tRNA-dependent Cys biosynthesis machinery, resulting in the co-occurrence of up to four unique genetic code systems (Figure 5; Sauerwald et al., 2005; Fukunaga and Yokoyama, 2007). The coexistence of the three different tRNA-dependent amino acid biosynthesis routes within a singular organism is likely facilitated by the fact that all three systems are believed to have been present in LUCA.

## Outlook

These diverse archaeal genetic code systems have been known to exist for over a decade, if not much longer. However, the exact mechanisms involved for biosynthesis and regulation for some of these coding systems have yet to be elucidated (Yuan et al., 2010b; Mukai et al., 2021). The archaeal domain was the last domain of life to be recognized, therefore new archaeal species are still being discovered owing to advances in technology and bioinformatics (Tahon et al., 2021). The most widely used method for discovery and characterization of archaeal lineages is still through rRNA diversity surveys by amplifying the universal 16S rRNA gene (Baker et al., 2020). The non-specific primers used in these studies often fail to target the diversity of archaeal genomes (Baker et al., 2006). Instead, methods like genomic recovery and reconstruction, through metagenomic assembly and binning, have exposed archaeal genomes which had been overlooked (Baker et al., 2020). Additionally, single-cell genome sequencing has been used to access the genetic information of uncultivated archaeal lineages (Rinke et al., 2013). Single-cell genomics is also useful in analyzing the diversity in complex archaeal populations, as fine-scale heterogeneity can be observed from the genomes of individual cells, which is lost by other genomic techniques that composite data from multiple cells or strains (Blainey, 2013). These new strategies have caused an expansion of the archaeal phylogenetic tree and continued data mining will cause constant growth, to fill in current gaps in knowledge about archaeal translation.

Additionally, studying the unique genetic code systems in archaeal lends itself to applications in genetic code expansion, taking advantage of the natural stop codon recoding in these organisms. The Pyl system has already been readily adapted for the incorporation of noncanonical amino acids (ncAAs) at diverse codons given its natural orthogonality to endogenous aaRS/tRNA pairs in prokaryotes and eukaryotes, the anticodon-independent recognition of tRNA^Pyl^, and the ability of the PylRS active site to accept non-natural substrates (Dunkelmann et al., 2020). By introducing mutations to further increase the orthogonality between different classes of PylRS and tRNA^Pyl^, the Pyl system can be used to incorporate multiple ncAAs within the same peptide. The feasibility of this idea was demonstrated when HMET1 PylRS1/tRNA^Pyl^1 and PylRS2/tRNA^Pyl^2 were used simultaneously to incorporate two different ncAAs into a single protein (Zhang et al., 2022).

A greater understanding of archaeal genomes also serves to elucidate the evolutionary relationship between archaea, bacteria, and eukaryotes. Originally grouped with bacteria as single-celled organisms, the complexity of archaeal mechanisms and processes is similar to that of eukaryotes (Gribaldo and Brochier-Armanet, 2006). The analysis of archaeal lineages like *Lokiarchaeota* and its Sec machinery have contributed to illustrate the transition between archaea and eukaryotes by displaying characteristics of both domains (Mariotti et al., 2016). As more archaeal genomes are analyzed and compared with those of bacteria and eukaryotes, the gaps in the evolutionary tree will be filled.

Ultimately, dedicated research on archaea has only just begun, and much is still left to be explored. In particular, the capacities of organisms in this domain to withstand a variety of extreme conditions have yet to be fully explained, though comparisons with the better understood mechanisms of these systems in bacteria or eukaryotes may inform understanding of the archaeal pathways. Analysis of the differences between organisms of these three domains may inspire strategies to engineer enhanced properties in bacteria and eukaryotes by borrowing natural archaeal machinery. Like the environments they inhabit, archaea possess a diverse range of applications, serving as a wealth of potential for future discovery and innovation.

## Author contributions

KM, CZC, and NK wrote the manuscript. NK conceptualized the manuscript. DS edited the manuscript. All authors contributed to the article and approved the submitted version.

## Funding

This work was supported by grants from the National Institute of General Medical Sciences (R35GM122560-05S1 to DS) and the Department of Energy Office of Basic Energy Sciences (DE-FG0298ER2031 to DS). CZC holds a Postdoctoral Fellowship from the Natural Sciences and Engineering Research Council of Canada (NSERC) and KM holds a Yale College Dean’s Research Fellowship from Yale University.

## Conflict of interest

The authors declare that the research was conducted in the absence of any commercial or financial relationships that could be construed as a potential conflict of interest.

## Publisher’s note

All claims expressed in this article are solely those of the authors and do not necessarily represent those of their affiliated organizations, or those of the publisher, the editors and the reviewers. Any product that may be evaluated in this article, or claim that may be made by its manufacturer, is not guaranteed or endorsed by the publisher.

## References

[ref1] Alkalaeva E. Eliseev B. Ambrogelly A. Vlasov P. Kondrashov F. A. Gundllapalli S.. (2009). Translation termination in pyrrolysine-utilizing archaea. FEBS Lett. 583, 3455–3460. doi: 10.1016/j.febslet.2009.09.044, PMID: 19796638 PMC2857517

[ref2] Ambrogelly A. Gundllapalli S. Herring S. Polycarpo C. Frauer C. Söll D. (2007). Pyrrolysine is not hardwired for cotranslational insertion at UAG codons. Proc. Natl. Acad. Sci. U. S. A. 104, 3141–3146. doi: 10.1073/pnas.0611634104, PMID: 17360621 PMC1805618

[ref3] Arnez J. G. Moras D. (1997). Structural and functional considerations of the aminoacylation reaction. Trends Biochem. Sci. 22, 211–216. doi: 10.1016/S0968-0004(97)01052-9, PMID: 9204708

[ref4] Bailly M. Blaise M. Lorber B. Becker H. D. Kern D. (2007). The transamidosome: A dynamic ribonucleoprotein particle dedicated to prokaryotic tRNA-dependent asparagine biosynthesis. Mol. Cell 28, 228–239. doi: 10.1016/j.molcel.2007.08.017, PMID: 17964262

[ref5] Baker B. J. De Anda V. Seitz K. W. Dombrowski N. Santoro A. E. Lloyd K. G. (2020). Diversity, ecology and evolution of Archaea. Nat. Microbiol. 5, 887–900. doi: 10.1038/s41564-020-0715-z, PMID: 32367054

[ref6] Baker B. J. Tyson G. W. Webb R. I. Flanagan J. Hugenholtz P. Allen E. E.. (2006). Lineages of acidophilic archaea revealed by community genomic analysis. Science 314, 1933–1935. doi: 10.1126/science.1132690, PMID: 17185602

[ref7] Baumann T. Exner M. Budisa N. (2018). Orthogonal protein translation using pyrrolysyl-tRNA synthetases for single- and multiple-noncanonical amino acid mutagenesis. Adv. Biochem. Eng. Biotechnol. 162, 1–19. doi: 10.1007/10_2016_37, PMID: 27783132

[ref8] Becker H. D. Reinbolt J. Kreutzer R. Giegé R. Kern D. (1997). Existence of two distinct aspartyl-tRNA synthetases in *Thermus thermophilus*. Structural and biochemical properties of the two enzymes. Biochemistry 36, 8785–8797. doi: 10.1021/bi970392v, PMID: 9220965

[ref9] Blainey P. C. (2013). The future is now: single-cell genomics of bacteria and archaea. FEMS Microbiol. Rev. 37, 407–427. doi: 10.1111/1574-6976.12015, PMID: 23298390 PMC3878092

[ref10] Blight S. K. Larue R. C. Mahapatra A. Longstaff D. G. Chang E. Zhao G.. (2004). Direct charging of tRNA^CUA^ with pyrrolysine *in vitro* and *in vivo*. Nature 431, 333–335. doi: 10.1038/nature02895, PMID: 15329732

[ref11] Borrel G. Gaci N. Peyret P. O'Toole P. W. Gribaldo S. Brugère J.-F. (2014). Unique characteristics of the pyrrolysine system in the 7th order of methanogens: implications for the evolution of a genetic code expansion cassette. Archaea 2014, 374146. doi: 10.1155/2014/374146, PMID: 24669202 PMC3941956

[ref12] Borrel G. Harris H. M. B. Tottey W. Mihajlovski A. Parisot N. Peyretaillade E.. (2012). Genome sequence of “*Candidatus* Methanomethylophilus alvus” mx 1201, a methanogenic archaeon from the human gut belonging to a seventh order of methanogens. J. Bacteriol. 194, 6944–6945. doi: 10.1128/jb.01867-12, PMID: 23209209 PMC3510639

[ref13] Brugère J.-F. Atkins J. F. O’Toole P. W. Borrel G. (2018). Pyrrolysine in archaea: a 22^nd^ amino acid encoded through a genetic code expansion. Emerg. Top. Life. Sci. 2, 607–618. doi: 10.1042/etls20180094, PMID: 33525836

[ref14] Bult C. J. White O. Olsen G. J. Zhou L. Fleischmann R. D. Sutton G. G.. (1996). Complete genome sequence of the methanogenic archaeon. Methanococcus jannaschii. Science 273, 1058–1073. doi: 10.1126/science.273.5278.1058 8688087

[ref15] Carlson B. A. Xu X. M. Kryukov G. V. Rao M. Berry M. J. Gladyshev V. N.. (2004). Identification and characterization of phosphoseryl-tRNA^[Ser]Sec^ kinase. Proc. Natl. Acad. Sci. U. S. A. 101, 12848–12853. doi: 10.1073/pnas.0402636101, PMID: 15317934 PMC516484

[ref16] Chen M. Kato K. Kubo Y. Tanaka Y. Liu Y. Long F.. (2017). Structural basis for tRNA-dependent cysteine biosynthesis. Nat. Commun. 8, 1521. doi: 10.1038/s41467-017-01543-y, PMID: 29142195 PMC5688128

[ref17] Cheng L. Qiu T. L. Yin X. B. Wu X. L. Hu G. Q. Deng Y.. (2007). *Methermicoccus shengliensis* gen. Nov., sp. nov., a thermophilic, methylotrophic methanogen isolated from oil-production water, and proposal of *Methermicoccaceae* fam. Nov. Int. J. Syst. Evol. Microbiol. 57, 2964–2969. doi: 10.1099/ijs.0.65049-0, PMID: 18048758

[ref18] Cone J. E. Río R. M. D. Davis J. N. Stadtman T. C. (1976). Chemical characterization of the selenoprotein component of clostridial glycine reductase: identification of selenocysteine as the organoselenium moiety. Proc. Natl. Acad. Sci. U. S. A. 73, 2659–2663. doi: 10.1073/pnas.73.8.2659, PMID: 1066676 PMC430707

[ref19] Curnow A. W. Ibba M. Söll D. (1996). tRNA-dependent asparagine formation. Nature 382, 589–590. doi: 10.1038/382589b0 8757127

[ref20] Dunkelmann D. L. Willis J. C. W. Beattie A. T. Chin J. W. (2020). Engineered triply orthogonal pyrrolysyl–tRNA synthetase/tRNA pairs enable the genetic encoding of three distinct non-canonical amino acids. Nat. Chem. 12, 535–544. doi: 10.1038/s41557-020-0472-x, PMID: 32472101 PMC7116526

[ref21] Eriani G. Delarue M. Poch O. Gangloff J. Moras D. (1990). Partition of tRNA synthetases into two classes based on mutually exclusive sets of sequence motifs. Nature 347, 203–206. doi: 10.1038/347203a0, PMID: 2203971

[ref22] Evans R. M. Krahn N. Murphy B. J. Lee H. Armstrong F. A. Söll D. (2021). Selective cysteine-to-selenocysteine changes in a [NiFe]-hydrogenase confirm a special position for catalysis and oxygen tolerance. Proc. Natl. Acad. Sci. U. S. A. 118:e2100921118. doi: 10.1073/pnas.2100921118, PMID: 33753519 PMC8020662

[ref23] Fagegaltier D. Carbon P. Krol A. (2001). Distinctive features in the Sel B family of elongation factors for selenoprotein synthesis. A glimpse of an evolutionary complexified translation apparatus. Bio Factors 14, 5–10. doi: 10.1002/biof.5520140102 11568434

[ref24] Fagegaltier D. Hubert N. Yamada K. Mizutani T. Carbon P. Krol A. (2000). Characterization of mSelB, a novel mammalian elongation factor for selenoprotein translation. EMBO J. 19, 4796–4805. doi: 10.1093/emboj/19.17.4796, PMID: 10970870 PMC302067

[ref25] Fukunaga R. Yokoyama S. (2007). Structural insights into the first step of RNA-dependent cysteine biosynthesis in archaea. Nat. Struct. Mol. Biol. 14, 272–279. doi: 10.1038/nsmb1219, PMID: 17351629

[ref26] Garcia G. E. Stadtman T. C. (1992). *Clostridium sticklandii* glycine reductase selenoprotein A gene: cloning, sequencing, and expression in *Escherichia coli*. J. Bacteriol. 174, 7080–7089. doi: 10.1128/jb.174.22.7080-7089.1992, PMID: 1429431 PMC207396

[ref27] Gaston M. A. Jiang R. Krzycki J. A. (2011a). Functional context, biosynthesis, and genetic encoding of pyrrolysine. Curr. Opin. Microbiol. 14, 342–349. doi: 10.1016/j.mib.2011.04.001, PMID: 21550296 PMC3119745

[ref28] Gaston M. A. Zhang L. Green-Church K. B. Krzycki J. A. (2011b). The complete biosynthesis of the genetically encoded amino acid pyrrolysine from lysine. Nature 471, 647–650. doi: 10.1038/nature09918, PMID: 21455182 PMC3070376

[ref29] Giegé R. Sissler M. Florentz C. (1998). Universal rules and idiosyncratic features in tRNA identity. Nucleic Acids Res. 26, 5017–5035. doi: 10.1093/nar/26.22.5017, PMID: 9801296 PMC147952

[ref30] Gribaldo S. Brochier-Armanet C. (2006). The origin and evolution of Archaea: a state of the art. Philos. Trans. R. Soc. Lond. Ser. B Biol. Sci. 361, 1007–1022. doi: 10.1098/rstb.2006.1841, PMID: 16754611 PMC1578729

[ref31] Guo L.-T. Amikura K. Jiang H.-K. Mukai T. Fu X. Wang Y.-S.. (2022). Ancestral archaea expanded the genetic code with pyrrolysine. J. Biol. Chem. 10.1016/j.jbc.2022.102521 PMC9630628 36152750

[ref32] Hao B. Gong W. Ferguson T. K. James C. M. Krzycki J. A. Chan M. K. (2002). A new UAG-encoded residue in the structure of a methanogen methyltransferase. Science 296, 1462–1466. doi: 10.1126/science.1069556, PMID: 12029132

[ref33] Hemmerle M. Wendenbaum M. Grob G. Yakobov N. Mahmoudi N. Senger B.. (2020). “Noncanonical inputs and outputs of tRNA aminoacylation” in The Enzymes. eds. Pouplana R. D. Kaguni L. S. (London: Academic Press), 117–147. 10.1016/bs.enz.2020.04.003 33837702

[ref34] Herring S. Ambrogelly A. Gundllapalli S. O’Donoghue P. Polycarpo C. R. Söll D. (2007). The amino-terminal domain of pyrrolysyl-tRNA synthetase is dispensable *in vitro* but required for *in vivo* activity. FEBS Lett. 581, 3197–3203. doi: 10.1016/j.febslet.2007.06.004, PMID: 17582401 PMC2074874

[ref35] Huot J. L. Balg C. Jahn D. Moser J. Émond A. Blais S. P.. (2007). Mechanism of a gat CAB amidotransferase: Aspartyl-tRNA synthetase increases its affinity for asp-tRNA^Asn^ and novel aminoacyl-tRNA analogues are competitive inhibitors. Biochemistry 46, 13190–13198. doi: 10.1021/bi700602n, PMID: 17929881

[ref36] Itoh Y. Chiba S. Sekine S.-I. Yokoyama S. (2009). Crystal structure of human selenocysteine tRNA. Nucleic Acids Res. 37, 6259–6268. doi: 10.1093/nar/gkp648, PMID: 19692584 PMC2764427

[ref37] Jiang R. Krzycki J. A. (2012). Pyl Sn and the homologous N-terminal domain of pyrrolysyl-tRNA synthetase bind the tRNA that is essential for the genetic encoding of pyrrolysine. J. Biol. Chem. 287, 32738–32746. doi: 10.1074/jbc.M112.396754, PMID: 22851181 PMC3463324

[ref38] Kavran J. M. Gundllapalli S. O'Donoghue P. Englert M. Söll D. Steitz T. A. (2007). Structure of pyrrolysyl-tRNA synthetase, an archaeal enzyme for genetic code innovation. Proc. Natl. Acad. Sci. U. S. A. 104, 11268–11273. doi: 10.1073/pnas.0704769104, PMID: 17592110 PMC2040888

[ref39] Kinzy S. A. Caban K. Copeland P. R. (2005). Characterization of the SECIS binding protein 2 complex required for the co-translational insertion of selenocysteine in mammals. Nucleic Acids Res. 33, 5172–5180. doi: 10.1093/nar/gki826, PMID: 16155186 PMC1214547

[ref40] Krahn N. Tharp J. M. Crnković A. Söll D. (2020). Engineering aminoacyl-tRNA synthetases for use in synthetic biology. Enzyme 48, 351–395. doi: 10.1016/bs.enz.2020.06.004, PMID: 33837709 PMC8086897

[ref41] Krzycki J. A. (2013). The path of lysine to pyrrolysine. Curr. Opin. Chem. Biol. 17, 619–625. doi: 10.1016/j.cbpa.2013.06.023, PMID: 23856058

[ref42] Lapointe J. Duplain L. Proulx M. (1986). A single glutamyl-tRNA synthetase aminoacylates tRNA^Glu^ and tRNA^Gln^ in *Bacillus subtilis* and efficiently misacylates *Escherichia coli* tRNA^Gln^_1_ *in vitro*. J. Bacteriol. 165, 88–93. doi: 10.1128/jb.165.1.88-93.1986, PMID: 3079749 PMC214374

[ref43] Latrèche L. Jean-Jean O. Driscoll D. M. Chavatte L. (2009). Novel structural determinants in human SECIS elements modulate the translational recoding of UGA as selenocysteine. Nucleic Acids Res. 37, 5868–5880. doi: 10.1093/nar/gkp635, PMID: 19651878 PMC2761289

[ref44] Leibundgut M. Frick C. Thanbichler M. Böck A. Ban N. (2005). Selenocysteine tRNA-specific elongation factor Sel B is a structural chimaera of elongation and initiation factors. EMBO J. 24, 11–22. doi: 10.1038/sj.emboj.7600505, PMID: 15616587 PMC544917

[ref45] Liu Y. Nakamura A. Nakazawa Y. Asano N. Ford K. A. Hohn M. J.. (2014). Ancient translation factor is essential for tRNA-dependent cysteine biosynthesis in methanogenic archaea. Proc. Natl. Acad. Sci. U. S. A. 111, 10520–10525. doi: 10.1073/pnas.1411267111, PMID: 25002468 PMC4115501

[ref46] Liu Y. Vinyard D. J. Reesbeck M. E. Suzuki T. Manakongtreecheep K. Holland P. L.. (2016). A [3Fe-4S] cluster is required for tRNA thiolation in archaea and eukaryotes. Proc. Natl. Acad. Sci. U. S. A. 113, 12703–12708. doi: 10.1073/pnas.1615732113, PMID: 27791189 PMC5111681

[ref47] Makino Y. Sato T. Kawamura H., Hachisuka, S.-i., Takeno R. Imanaka T.. (2016). An archaeal ADP-dependent serine kinase involved in cysteine biosynthesis and serine metabolism. Nat. Commun. 7, 13446. doi:10.1038/ncomms13446, PMID: . 27857065 PMC5120207

[ref48] Mallick B. Chakrabarti J. Sahoo S. Ghosh Z. Das S. (2005). Identity elements of archaeal tRNA. DNA Res. 12, 235–246. doi: 10.1093/dnares/dsi008, PMID: 16769686

[ref49] Marck C. Grosjean H. (2002). tRNomics: analysis of tRNA genes from 50 genomes of Eukarya, Archaea, and bacteria reveals anticodon-sparing strategies and domain-specific features. RNA 8, 1189–1232. doi: 10.1017/S1355838202022021, PMID: 12403461 PMC1370332

[ref50] Mariotti M. Lobanov A. V. Manta B. Santesmasses D. Bofill A. Guigó R.. (2016). Lokiarchaeota marks the transition between the archaeal and eukaryotic selenocysteine encoding systems. Mol. Biol. Evol. 33, 2441–2453. doi: 10.1093/molbev/msw122, PMID: 27413050 PMC4989117

[ref51] Mukai T. Amikura K. Fu X. Söll D. Crnković A. (2021). Indirect routes to aminoacyl-tRNA: The diversity of prokaryotic cysteine encoding systems. Front. Genet. 12:794509. doi: 10.3389/fgene.2021.794509, PMID: 35047015 PMC8762117

[ref52] Mukai T. Crnković A. Umehara T. Ivanova N. N. Kyrpides N. C. Söll D.. (2017). RNA-dependent cysteine biosynthesis in bacteria and archaea. MBio 8, e00561–e00517. doi: 10.1128/mBio.00561-17, PMID: 28487430 PMC5424206

[ref53] Mwirichia R. Alam I. Rashid M. Vinu M. Ba-Alawi W. Anthony Kamau A.. (2016). Metabolic traits of an uncultured archaeal lineage—MSBL1—from brine pools of the Red Sea. Sci. Rep. 6, 19181. doi: 10.1038/srep19181, PMID: 26758088 PMC4725937

[ref54] Namgoong S. Sheppard K. Sherrer R. L. Söll D. (2007). Co-evolution of the archaeal tRNA-dependent amidotransferase Gat CAB with tRNA^Asn^. FEBS Lett. 581, 309–314. doi: 10.1016/j.febslet.2006.12.033, PMID: 17214986 PMC1808439

[ref55] Nozawa K. O'Donoghue P. Gundllapalli S. Araiso Y. Ishitani R. Umehara T.. (2009). Pyrrolysyl-tRNA synthetase-tRNA^Pyl^ structure reveals the molecular basis of orthogonality. Nature 457, 1163–1167. doi: 10.1038/nature07611, PMID: 19118381 PMC2648862

[ref56] O'Donoghue P. Sethi A. Woese C. R. Luthey-Schulten Z. A. (2005). The evolutionary history of Cys-tRNA^Cys^ formation. Proc. Natl. Acad. Sci. U. S. A. 102, 19003–19008. doi: 10.1073/pnas.0509617102, PMID: 16380427 PMC1323144

[ref57] Oshikane H. Sheppard K. Fukai S. Nakamura Y. Ishitani R. Numata T.. (2006). Structural basis of RNA-dependent recruitment of glutamine to the genetic code. Science 312, 1950–1954. doi: 10.1126/science.1128470, PMID: 16809540

[ref58] Patteson K. G. Trivedi N. Stadtman T. C. (2005). *Methanococcus vannielii* selenium-binding protein (SeBP): chemical reactivity of recombinant SeBP produced in *Escherichia coli*. Proc. Natl. Acad. Sci. U. S. A. 102, 12029–12034. doi: 10.1073/pnas.0505650102, PMID: 16103372 PMC1189349

[ref59] Poehlein A. Heym D. Quitzke V. Fersch J. Daniel R. Rother M. (2018). Complete genome sequence of the *Methanococcus maripaludis* type strain JJ (DSM 2067), a model for selenoprotein synthesis in archaea. Genome Announc. 6, e00237–e00218. doi: 10.1128/genomeA.00237-18, PMID: 29622618 PMC5887029

[ref60] Rampias T. Sheppard K. Söll D. (2010). The archaeal transamidosome for RNA-dependent glutamine biosynthesis. Nucleic Acids Res. 38, 5774–5783. doi: 10.1093/nar/gkq336, PMID: 20457752 PMC2943598

[ref61] Reed C. J. Lewis H. Trejo E. Winston V. Evilia C. (2013). Protein adaptations in archaeal extremophiles. Archaea 2013:373275. doi: 10.1155/2013/373275, PMID: 24151449 PMC3787623

[ref62] Rinke C. Schwientek P. Sczyrba A. Ivanova N. N. Anderson I. J. Cheng J.-F.. (2013). Insights into the phylogeny and coding potential of microbial dark matter. Nature 499, 431–437. doi: 10.1038/nature12352, PMID: 23851394

[ref63] Rother M. Krzycki J. A. (2010). Selenocysteine, pyrrolysine, and the unique energy metabolism of methanogenic archaea. Archaea 2010:453642. doi: 10.1155/2010/453642, PMID: 20847933 PMC2933860

[ref64] Rother M. Quitzke V. (2018). Selenoprotein synthesis and regulation in archaea. Biochim. Biophys. Acta, Gen. Subj. 1862, 2451–2462. doi: 10.1016/j.bbagen.2018.04.008, PMID: 29656122

[ref65] Rother M. Resch A. Wilting R. Böck A. (2001). Selenoprotein synthesis in archaea. Bio Factors 14, 75–83. doi: 10.1002/biof.5520140111 11568443

[ref66] Rother M. Wilting R. Commans S. Böck A. (2000). Identification and characterisation of the selenocysteine-specific translation factor Sel B from the archaeon *Methanococcus jannaschii*. J. Mol. Biol. 299, 351–358. doi: 10.1006/jmbi.2000.3756, PMID: 10860743

[ref67] Rothschild L. J. Mancinelli R. L. (2001). Life in extreme environments. Nature 409, 1092–1101. doi: 10.1038/35059215 11234023

[ref68] Santesmasses D. Mariotti M. Guigó R. (2017). Computational identification of the selenocysteine tRNA (tRNA^Sec^) in genomes. PLoS Comput. Biol. 13:e1005383. doi: 10.1371/journal.pcbi.1005383, PMID: 28192430 PMC5330540

[ref69] Sauerwald A. Zhu W. Major T. A. Roy H. Palioura S. Jahn D.. (2005). RNA-dependent cysteine biosynthesis in archaea. Science 307, 1969–1972. doi: 10.1126/science.1108329, PMID: 15790858

[ref70] Schön A. Böck A. Ott G. Söll D. (1989). The selenocysteine-inserting opal suppressor serine tRNA from E. coli is highly unusual in structure and modification. Nucleic Acids Res. 17, 7159–7165. doi: 10.1093/nar/17.18.7159, PMID: 2529478 PMC334795

[ref71] Schön A. Kannangara C. G. Gough S. Söll D. (1988). Protein biosynthesis in organelles requires misaminoacylation of tRNA. Nature 331, 187–190. doi: 10.1038/331187a0, PMID: 3340166

[ref72] Self W. Pierce R. Stadtman T. (2004). Cloning and heterologous expression of a *Methanococcus vannielii* gene encoding a selenium-binding protein. IUBMB Life 56, 501–507. doi: 10.1080/15216540400010818, PMID: 15545230

[ref73] Selmer M. Su X. D. (2002). Crystal structure of an mRNA-binding fragment of *Moorella thermoacetica* elongation factor Sel B. EMBO J. 21, 4145–4153. doi: 10.1093/emboj/cdf408, PMID: 12145214 PMC126154

[ref74] Sheppard K. Sherrer R. L. Söll D. (2008a). *Methanothermobacter thermautotrophicus* tRNA^Gln^ confines the amidotransferase Gat CAB to asparaginyl-tRNA^Asn^ formation. J. Mol. Biol. 377, 845–853. doi: 10.1016/j.jmb.2008.01.064, PMID: 18291416 PMC2390905

[ref75] Sheppard K. Söll D. (2008). On the evolution of the tRNA-dependent amidotransferases, Gat CAB and Gat DE. J. Mol. Biol. 377, 831–844. doi: 10.1016/j.jmb.2008.01.016, PMID: 18279892 PMC2366055

[ref76] Sheppard K. Yuan J. Hohn M. J. Jester B. Devine K. M. Söll D. (2008b). From one amino acid to another: tRNA-dependent amino acid biosynthesis. Nucleic Acids Res. 36, 1813–1825. doi: 10.1093/nar/gkn015, PMID: 18252769 PMC2330236

[ref77] Sherrer R. L. Araiso Y. Aldag C. Ishitani R. Ho J. M. L. Söll D.. (2010). C-terminal domain of archaeal *O*-phosphoseryl-tRNA kinase displays large-scale motion to bind the 7-bp D-stem of archaeal tRNA^Sec^. Nucleic Acids Res. 39, 1034–1041. doi: 10.1093/nar/gkq845, PMID: 20870747 PMC3035459

[ref78] Sherrer R. L. Ho J. M. L. Söll D. (2008a). Divergence of selenocysteine tRNA recognition by archaeal and eukaryotic *O*-phosphoseryl-tRNA^Sec^ kinase. Nucleic Acids Res. 36, 1871–1880. doi: 10.1093/nar/gkn036, PMID: 18267971 PMC2330242

[ref79] Sherrer R. L. O'Donoghue P. Söll D. (2008b). Characterization and evolutionary history of an archaeal kinase involved in selenocysteinyl-tRNA formation. Nucleic Acids Res. 36, 1247–1259. doi: 10.1093/nar/gkm1134, PMID: 18174226 PMC2275090

[ref80] Srinivasan G. James C. M. Krzycki J. A. (2002). Pyrrolysine encoded by UAG in archaea: charging of a UAG-decoding specialized tRNA. Science 296, 1459–1462. doi: 10.1126/science.1069588, PMID: 12029131

[ref81] Stock T. Rother M. (2009). Selenoproteins in Archaea and gram-positive bacteria. Biochim. Biophys. Acta, Gen. Subj. 1790, 1520–1532. doi: 10.1016/j.bbagen.2009.03.022, PMID: 19344749

[ref82] Sturchler C. Westhof E. Carbon P. Krol A. (1993). Unique secondary and tertiary structural features of the eucaryotic selenocysteine tRNA^Sec^. Nucleic Acids Res. 21, 1073–1079. doi: 10.1093/nar/21.5.1073, PMID: 8464694 PMC309265

[ref83] Sun J. Evans P. N. Gagen E. J. Woodcroft B. J. Hedlund B. P. Woyke T.. (2021). Recoding of stop codons expands the metabolic potential of two novel Asgardarchaeota lineages. ISME Commun. 1, 30. doi: 10.1038/s43705-021-00032-0 PMC9723677 36739331

[ref84] Suzuki T. Miller C. Guo L.-T. Ho J. M. L. Bryson D. I. Wang Y.-S.. (2017). Crystal structures reveal an elusive functional domain of pyrrolysyl-tRNA synthetase. Nat. Chem. Biol. 13, 1261–1266. doi: 10.1038/nchembio.2497, PMID: 29035363 PMC5698177

[ref85] Suzuki T. Nakamura A. Kato K. Söll D. Tanaka I. Sheppard K.. (2015). Structure of the *Pseudomonas aeruginosa* transamidosome reveals unique aspects of bacterial tRNA-dependent asparagine biosynthesis. Proc. Natl. Acad. Sci. U. S. A. 112, 382–387. doi: 10.1073/pnas.1423314112, PMID: 25548166 PMC4299244

[ref86] Tahon G. Geesink P. Ettema T. J. G. (2021). Expanding archaeal diversity and phylogeny: past, present, and future. Annu. Rev. Microbiol. 75, 359–381. doi: 10.1146/annurev-micro-040921-050212, PMID: 34351791

[ref87] Tharp J. M. Ehnbom A. Liu W. R. (2018). tRNA^Pyl^: structure, function, and applications. RNA Biol. 15, 441–452. doi: 10.1080/15476286.2017.1356561, PMID: 28837402 PMC6103707

[ref88] Tumbula D. L. Becker H. D. Chang W.-Z. Söll D. (2000). Domain-specific recruitment of amide amino acids for protein synthesis. Nature 407, 106–110. doi: 10.1038/35024120, PMID: 10993083

[ref89] Turanov A. A. Lobanov A. V. Fomenko D. E. Morrison H. G. Sogin M. L. Klobutcher L. A.. (2009). Genetic code supports targeted insertion of two amino acids by one codon. Science 323, 259–261. doi: 10.1126/science.1164748, PMID: 19131629 PMC3088105

[ref90] Wan W. Tharp J. M. Liu W. R. (2014). Pyrrolysyl-tRNA synthetase: an ordinary enzyme but an outstanding genetic code expansion tool. Biochim. Biophys. Acta 1844, 1059–1070. doi: 10.1016/j.bbapap.2014.03.002, PMID: 24631543 PMC4016821

[ref91] Whittaker R. H. (1969). New concepts of kingdoms of organisms. Science 163, 150–160. doi: 10.1126/science.163.3863.150, PMID: 5762760

[ref92] Wilcox M. Nirenberg M. (1968). Transfer RNA as a cofactor coupling amino acid synthesis with that of protein. Proc. Natl. Acad. Sci. U. S. A. 61, 229–236. doi: 10.1073/pnas.61.1.229, PMID: 4972364 PMC285927

[ref93] Willis J. C. W. Chin J. W. (2018). Mutually orthogonal pyrrolysyl-tRNA synthetase/tRNA pairs. Nat. Chem. 10, 831–837. doi: 10.1038/s41557-018-0052-5, PMID: 29807989 PMC6055992

[ref94] Wilting R. Schorling S. Persson B. C. Böck A. (1997). Selenoprotein synthesis in archaea: identification of an mRNA element of *Methanococcus jannaschii* probably directing selenocysteine insertion. J. Mol. Biol. 266, 637–641. doi: 10.1006/jmbi.1996.0812, PMID: 9102456

[ref95] Woese C. R. Fox G. E. Zablen L. Uchida T. Bonen L. Pechman K.. (1975). Conservation of primary structure in 16S ribosomal RNA. Nature 254, 83–86. doi: 10.1038/254083a0, PMID: 1089909

[ref96] Woese C. R. Olsen G. J. Ibba M. Söll D. (2000). Aminoacyl-tRNA synthetases, the genetic code, and the evolutionary process. Microbiol. Mol. Biol. Rev. 64, 202–236. doi: 10.1128/MMBR.64.1.202-236.2000, PMID: 10704480 PMC98992

[ref97] Xu X.-M. Turanov A. A. Carlson B. A. Yoo M.-H. Everley R. A. Nandakumar R.. (2010). Targeted insertion of cysteine by decoding UGA codons with mammalian selenocysteine machinery. Proc. Natl. Acad. Sci. U. S. A. 107, 21430–21434. doi: 10.1073/pnas.1009947107, PMID: 21115847 PMC3003055

[ref98] Yuan J. Hohn M. J. Sherrer R. L. Palioura S. Su D. Söll D. (2010a). A tRNA-dependent cysteine biosynthesis enzyme recognizes the selenocysteine-specific tRNA in *Escherichia coli*. FEBS Lett. 584, 2857–2861. doi: 10.1016/j.febslet.2010.05.028, PMID: 20493852 PMC2893348

[ref99] Yuan J. O'Donoghue P. Ambrogelly A. Gundllapalli S. Sherrer R. L. Palioura S.. (2010b). Distinct genetic code expansion strategies for selenocysteine and pyrrolysine are reflected in different aminoacyl-tRNA formation systems. FEBS Lett. 584, 342–349. doi: 10.1016/j.febslet.2009.11.005, PMID: 19903474 PMC2795046

[ref100] Zavacki A. M. Mansell J. B. Chung M. Klimovitsky B. Harney J. W. Berry M. J. (2003). Coupled tRNA^Sec^-dependent assembly of the selenocysteine decoding apparatus. Mol. Cell 11, 773–781. doi: 10.1016/s1097-2765(03)00064-9, PMID: 12667458

[ref101] Zhang Y. Baranov P. V. Atkins J. F. Gladyshev V. N. (2005). Pyrrolysine and selenocysteine use dissimilar decoding strategies. J. Biol. Chem. 280, 20740–20751. doi: 10.1074/jbc.M501458200, PMID: 15788401

[ref102] Zhang H. Gong X. Zhao Q. Mukai T. Vargas-Rodriguez O. Zhang H.. (2022). The tRNA discriminator base defines the mutual orthogonality of two distinct pyrrolysyl-tRNA synthetase/tRNA^Pyl^ pairs in the same organism. Nucleic Acids Res. 50, 4601–4615. doi: 10.1093/nar/gkac271, PMID: 35466371 PMC9071458

[ref103] Zhang H. Y. Qin T. Jiang Y. Y. Caetano-Anollés G. (2012). Structural phylogenomics uncovers the early and concurrent origins of cysteine biosynthesis and iron-sulfur proteins. J. Biomol. Struct. Dyn. 30, 542–545. doi: 10.1080/07391102.2012.687520, PMID: 22731683

[ref104] Zinoni F. Birkmann A. Stadtman T. C. Böck A. (1986). Nucleotide sequence and expression of the selenocysteine-containing polypeptide of formate dehydrogenase (formate-hydrogen-lyase-linked) from *Escherichia coli*. Proc. Natl. Acad. Sci. U. S. A. 83, 4650–4654. doi: 10.1073/pnas.83.13.4650, PMID: 2941757 PMC323799

